# Androgen receptor-dependent regulation of metabolism in high grade bladder cancer cells

**DOI:** 10.1038/s41598-023-28692-z

**Published:** 2023-01-31

**Authors:** Kimberley D. Katleba, Maria-Malvina Tsamouri, Maitreyee Jathal, Han Bit Baek, Rebecca B. Armenta, Clifford G. Tepper, Gino Cortopassi, Paramita M. Ghosh, Maria Mudryj

**Affiliations:** 1 Veterans Affairs-Northern California Health Care System, Mather, CA USA; 2 Department of Medical Microbiology and Immunology, University of California, 1 Shields Avenue, UC Davis, Davis, CA 95616 USA; 3 Department of Urologic Surgery, University of California, 1 Shields Avenue, UC Davis, Davis, CA 95616 USA; 4 Biochemistry and Molecular Medicine, University of California, 1 Shields Avenue, UC Davis, Davis, CA 95616 USA; 5 Davis Cancer Comprehensive Cancer Center, School of Medicine, University of California, 1 Shields Avenue, Tupper Hall, UC Davis, Davis, CA 95616 USA; 6 Department of Molecular Sciences, School of Veterinary Medicine, University of California, UC Davis, 1 Shields Avenue, Davis, CA 95616 USA

**Keywords:** Cancer, Oncology, Urology

## Abstract

The observed sex disparity in bladder cancer (BlCa) argues that androgen receptor (AR) signaling has a role in these malignancies. BlCas express full-length AR (FL-AR), constitutively active AR splice variants, including AR-v19, or both, and their depletion limits BlCa viability. However, the mechanistic basis of AR-dependence is unknown. Here, we depleted FL-AR, AR-v19, or all AR forms (T-AR), and performed RNA-seq studies to uncover that different AR forms govern distinct but partially overlapping transcriptional programs. Overlapping alterations include a decrease in mTOR and an increase of hypoxia regulated transcripts accompanied by a decline in oxygen consumption rate (OCR). Queries of BlCa databases revealed a significant negative correlation between AR expression and multiple hypoxia-associated transcripts arguing that this regulatory mechanism is a feature of high-grade malignancies. Our analysis of a 1600-compound library identified niclosamide as a strong ATPase inhibitor that reduces OCR in BlCa cells, decreased cell viability and induced apoptosis in a dose and time dependent manner. These results suggest that BlCa cells hijack AR signaling to enhance metabolic activity, promoting cell proliferation and survival; hence targeting this AR downstream vulnerability presents an attractive strategy to limit BlCa.

## Introduction

BlCa in the US accounted for over 83,000 new cases of cancer and ~ 17,200 deaths in 2021^1^. BlCa is classified as non-muscle invasive or muscle invasive and may be of high or low grade. Muscle invasive BlCa (MIBC) is most problematic and ultimately leads to fatalities^2^. BlCa displays a well-documented gender disparity where men are 3–4 times more likely to develop disease than women^3^. Numerous studies reported that the AR has a significant role underlying this disparity^4–6^. Immunohistochemical (IHC) analysis of BlCa tissue from males and females found that AR expression is higher in males^7^. Animal models confirmed that androgens contribute to bladder carcinogenesis. AR conditional expression in mouse urothelium and supplementation of female rats with testosterone promoted N-butyl-N-(4-hydroxybutyl) nitrosamine-induced tumor incidence while urothelium targeted AR knockout reduced tumor incidence^8,9^. Epidemiological studies found that men with BlCa treated with 5α-reductase inhibitors, which block conversion of testosterone to the more potent dihydrotestosterone, exhibited a lower risk of BlCa related death^10^, further supporting a role for AR in BlCa.

AR is a member of the 48-member steroid receptor superfamily, potent signaling molecules governing multiple cellular processes. All members share a basic structure consisting of activation and DNA binding regions linked to a ligand binding domain (LBD), conferring ligand-dependent regulation of transcription^11^. The AR normally exerts a regulatory role in multiple tissues including muscle, bone, brain, and prostate, but in malignancies this role can be perverted to benefit tumor cell proliferation and survival^12^. AR mediated androgen signaling is pivotal in prostatic malignancies, hence much of our knowledge on the mechanistic basis of AR signaling is derived from prostate cancer (PCa) studies. In PCa, the strong reliance of cells on AR signaling has been exploited in the development of therapeutics, which target the LBD to limit AR signaling, restricting tumor growth. Initially, these cancers respond to therapy, but eventually castration resistant malignancies emerge driven by multiple mechanisms, including aberrant splicing leading to expression of constitutively active low molecular weight (LMW) AR isoforms^13^ refractory to LBD targeting therapeutics.

We recently reported that while FL-AR is in some BlCa, most BlCa cells also express LMW splice variants. We identified a novel variant, AR-v19, which localizes to the nucleus, is expressed in multiple BlCa cell lines and tumors and transactivates transcription from an AR-regulated promoter in a dose dependent manner^14^. Importantly, siRNA mediated depletion of total-AR (T-AR) reduces viability and induces apoptosis in multiple BlCa cellular contexts^14^, confirming a dependence on AR signaling. Hence, limiting AR signaling is an attractive therapeutic strategy. However, the presence of constitutively active splice variants limits the efficacy of AR targeting drugs^14^. Given the accumulating evidence that the AR has a key role in BlCa tumorigenesis, but would be refractory to current therapies, defining AR downstream targets may identify actionable vulnerabilities.

Studies herein describe the transcriptomes regulated by depletion of T-AR, depletion of FL-AR or AR-v19, and cells overexpressing the AR-v19 splice variant. Key findings show that loss of all AR signaling alters oxidative phosphorylation components resulting in an elevation of HIF1A and its target genes, and a deregulation of pro and anti-apoptotic molecules. mTOR expression is repressed following depletion of all AR isoforms. Accordingly, depletion of AR alters mTOR downstream signaling and pharmacological targeting of mTOR effectively limits cell viability. Perusal of large high-grade BlCa datasets revealed that multiple HIF1A pathway components negatively correlate with AR, arguing that increased AR expression represses hypoxic responses. This aligns with our results that reduced AR signaling decreases OCR. Uncoupling oxidative phosphorylation with the antihelminth, niclosamide, was highly effective in reducing BlCa cell viability. Overall, these studies point to AR augmentation of metabolic processes as a targetable driver in AR-dependent BlCa.

## Material and methods

### Cell Culture, siRNA Transfections, Drug Treatment

UM-UC-3, TCCSUP, J82, T24, and RT4 were obtained from ATCC (Manassas, VA). All cell lines were cultured in RPMI-1640 supplemented with 10% fetal bovine serum (FBS), mmol/liter glutamine and 100 units/ml penicillin, and 100 ug/ml streptomycin (Invitrogen/Thermo Fisher Scientific; Waltham, MA) at 37 °C and 5% CO2. Cells used in studies were under passage 20 from the time they were received from ATCC. siRNA transfections were carried out using Lipofectamine RNAiMAX (Invitrogen) or PolyPlus (at an oligonucleotide concentration of 50 nM unless otherwise specified) maintained in the above media, and harvested 72 h post transfection, unless stated otherwise. The following oligonucleotides from Dharmacon (Thermo Scientific) were used for transfection; siAR-smart pool (L003400-00), control non-targeting oligonucleotide (D-001210–01), LBD targeting (J-003400–06)^15^, and AR-v19 specific siRNAs designed by us A(GGGAUGACUCUGGGAGGGUUUUU; B (GAUGACUCUGGGAGGGUCCUU)^14^. Niclosamide and sapanisertib were obtained from Selleck Chemicals (Houston, TX) and resuspended in DMSO.

### RNA extraction and qRT-PCR analysis

RNA and cDNA were prepared, and qPCR studies were conducted as previously described^14^. qRT-PCR was performed using the primers listed in Supplementary Fig. 1. Briefly, RNA was extracted using the RNeasy kit (Qiagen, 74,106) and cDNA was generated using QuantiTect (Qiagen; Germantown, MD) reverse transcription based on the manufacturer’s protocol. qRT PCR was performed using primers from Invitrogen. Standard curves were generated for each primer set using serial dilutions of cDNA to standardize variations in PCR reactions. In addition, after each run, melting curves were used to verify the melting temperature of the amplicon. All qPCR reactions were performed in triplicates. HPRT levels served as a quantification control. cDNAs were in ddH2O and added to QuantiTech SYBR Green PCR master mix (Qiagen) and 200 nM of each primer. PCR conditions had initial denaturation step at 95 C for 2 min 30 s, 40 cycles at 95 C for 13 s. Data were collected by the Mastercycler ep Realplex (Eppendorf AG, Hamburg, Germany).

### Cell lysates and western immunoblots

Cells were placed in a 4 °C RIPA lysis buffer containing a protease inhibitor mixture (Sigma). Thirty to fifty micrograms of protein were separated on 8%, 10%, or 12% SDS-PAGE gels, transferred to 0.45 µm nitrocellulose membranes (Thermo Scientific), and blocked with 5% nonfat dry milk in phosphate-buffered saline and 0.1% Tween 20 (PBS-T) or OneBlock Western-CL blocking buffer (Genesee Scientific; El Cajon, CA). If membranes were probed for more than one protein, then the membranes were cut prior to incubation with specific antibodies. Membranes were incubated with primary antibody overnight at 4C. The following antibodies were used: AR (N20), actin, GAPDH, cyclin A (239), E2F1 (251), and Cyclin D3 (182) (Santa Cruz Biotechnology; Santa Cruz, CA), AR A303 (Fortis, Waltham, MA) or A303 (Bethyl; Montgomery, TX), cleaved PARP, phospho-AKT (4060), phospho-p70S6K (9234), total AKT (4685), total p70S6K (9202), total mTOR (2972), cleaved PARP, and cyclin E (4129) (Cell Signaling; Danvers, MA). Tubulin, pAKT, and S70 (Thermo Scientific, Waltham, MA), The following day, membranes were washed with PBS-T 3 times, incubated with secondary antibody conjugated to HRP, and development was carried out using SuperSignal West Femto chemoluminescence (Thermo Scientific) or were imaged using LI-COR near-infrared western blot detection. Gel loading was assessed either by GAPDH or tubulin. Uncropped images are in Supplementary figures.

### Viability assays

Cells were plated at 10,000–20,000 cells/well in a 12 well plate and 24 h later treated with niclosamide, sapanisertib (Selleckchem), or DMSO control for 2 or 4 days. Viability was assessed using Cell Counting Kit – 8 (CCK-8) following manufacturer’s recommendations (Dojindo; Rockville, MD). For experiments assessing the growth of cells transfected with total AR siRNA, cells were plated in a 24 well plate at 10,000–20,000 cells/well and transfected 24 h later with a control oligonucleotide or AR targeting siRNA. Proliferation was assessed 72 h later using CCK-8 assay. All experiments were performed in triplicate. Data is displayed as the mean +/− standard deviation.

### Colony formation assays

Cells were plated in 6-well plates at 15,000 cells/well. The following day, cells were transfected with control or AR specific siRNA and were propagated for 10–12 days post transfection. Colonies were fixed and stained with crystal violet (0.5%) in 25% methanol/water, dried, and photographed. Image J was used to quantify the images.

### RNA-sequencing (RNA-Seq)

RNA was isolated and submitted to Novogene. RNA purity and concentration were measured using the NanoPhotometer Pearl (Implen). Following ribo-depletion, RNA-Seq libraries were prepared using a protocol to capture both mRNA and lncRNA transcripts and sequenced on an Illumina NovaSeq 6000 System (2 × 150 bp, paired-end). Data analysis was performed with a TopHat2-Cufflinks-Cuffdiff pipeline^16,17^ for mapping/alignment of raw sequence reads (FASTQ format) to the reference human genome assembly (GRCh37/hg19), transcript assembly, and quantitation of gene and transcript expression as FPKM (Fragments Per Kilobase of transcript per Million mapped reads). Data were annotated for unique genes/transcripts with Ensembl Release 82 (GRCh37/hg19). Group comparisons were conducted with Cuffdiff, and genes meeting a statistical threshold of an adjusted *P*-value < 0.05 were assigned as differentially expressed. Log2(fold change) and adjusted *P*-values were used as input for volcano plots, and normalized FPKM values utilized for hierarchical clustering and heatmap visualization. The ToppGene Suite^18^ and EnrichR^19^ platforms defined enrichment for molecular functions, biological processes, cellular components, and pathway alterations.

### Measure of oxygen consumption rate

Analyses were performed using an Agilent Seahorse XFe24 analyzer (Agilent Technologies). Cells were plated onto the Seahorse plate (Seahorse XF24 V7 PS Cell Culture Microplates, #100777-004) at 90,000 cells/well and cultured in 250 μL RPMI media (10% FBS and 1% Penicillin Streptomycin). Seahorse XF24 Sensor Cartridge was hydrated overnight using Seahorse XF Calibrant Solution (Seahorse XFe24 Flux Pak). Cells were placed in Seahorse XF RPMI assay medium supplemented with 10 mM glucose, 1 mM pyruvate, and 2 mM glutamine. Seahorse XF Cell Mito Stress Test Kit (#103,015–100,) was performed according to the manufacturer’s protocol, using the final well concentrations of 1.5 μM Oligomycin, 2 μM Carbonyl cyanide-4 (trifluoromethoxy) phenylhydrazone (FCCP) and 0.5 μM Rotenone/Antimycin A. On completion cells were counted using an Automatic cell counter (Countess II FL Cell Counter, Thermo Scientific Invitrogen) for data normalization. Data analysis used the Seahorse Wave (RRID:SCR_014526) Desktop software and was exported using the Seahorse XF Cell Mito Stress Test Report Generator.

### Statistics

For all assays,* a* two tailed two sample equal variance student’s t-test was used to assess differences between samples. For RT-qPCR experiments, the delta-delta Ct method was used to calculate fold change in gene expression as described before (https://www.sciencedirect.com/science/article/pii/S1046202301912629). A p < 0.05 was considered as statistically significant. Statistical analyses were preformed using GraphPad Prism 8.

## Results

### AR alterations in bladder malignancies are evident in ten percent of malignancies

**Figure 1:**
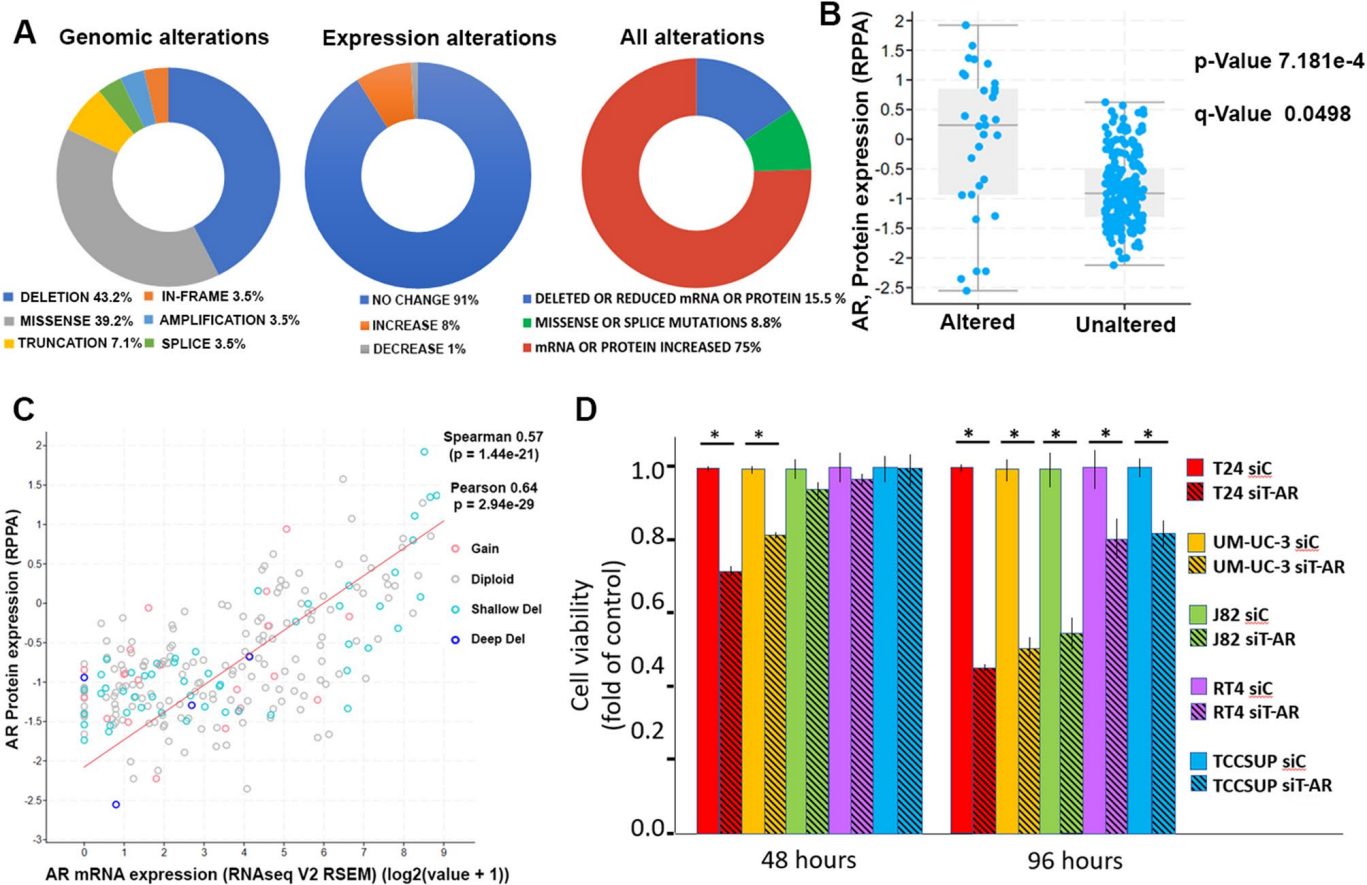
AR genomic and expression alterations in high grade BlCa. (**A**) AR alterations identified in bladder high grade malignancies (cBioPortal). Left panel- genetic alterations, middle panel- expression alterations, right panel—all alterations. (**B**) AR expression alterations correlated with increased AR levels. (**C**) Correlation between AR transcript and protein expression are statistically significant. (**D**) AR depletion reduces cell viability in multiple cells in a time dependent manner. The analysis was conducted in triplicate. *P-value < 0.05.

Two comprehensive studies on genetic and expression alterations in Muscle Invasive Bladder Cancer (LBD-AA), TCGA PanCancer Atlas and MSK TCGA 2020^20^, publicly available on cBioportal^21^ show that AR alterations are evident in 10–11% of bladder malignancies. AR gene alterations are present in 3% of malignancies, the most common being deletion or missense mutations (Fig. 1A, left panel). Most alterations are changes in transcript or protein expression where AR expression is decreased in 1% and elevated in 8% of profiled samples (Fig. 1A, middle panel), accounting for 75% of all AR alterations (Fig. 1A, right panel), therefore most tumors with AR alterations exhibit higher AR mRNA expression (Fig. 1B). Moreover, there is a strong correlation between AR transcript and protein levels (Fig. 1C), indicating that AR transcript elevation translates to an increase of AR protein. Multiple BlCa cells that are AR dependent exhibit a significant reduction in cell viability following T-AR depletion (Fig. 1D)^14^.

### Depletion of all AR reveals AR-dependent regulation of metabolism

UM-UC-3 which express FL-AR and the AR-v19 splice variant and show a strong response to total AR depletion (Fig. 1D), were utilized to identify transcript alterations following depletion of T-AR, FL-AR or the AR-v19 isoform. Following treatment with specific siRNAs cells were subjected to RNA-seq studies. To capture non-polyadenylated transcripts we employed a ribo-depletion strategy to enrich for regulated transcripts. Non-targeting siRNA served as a control and the studies were conducted using three biological replicates.

**Figure 2:**
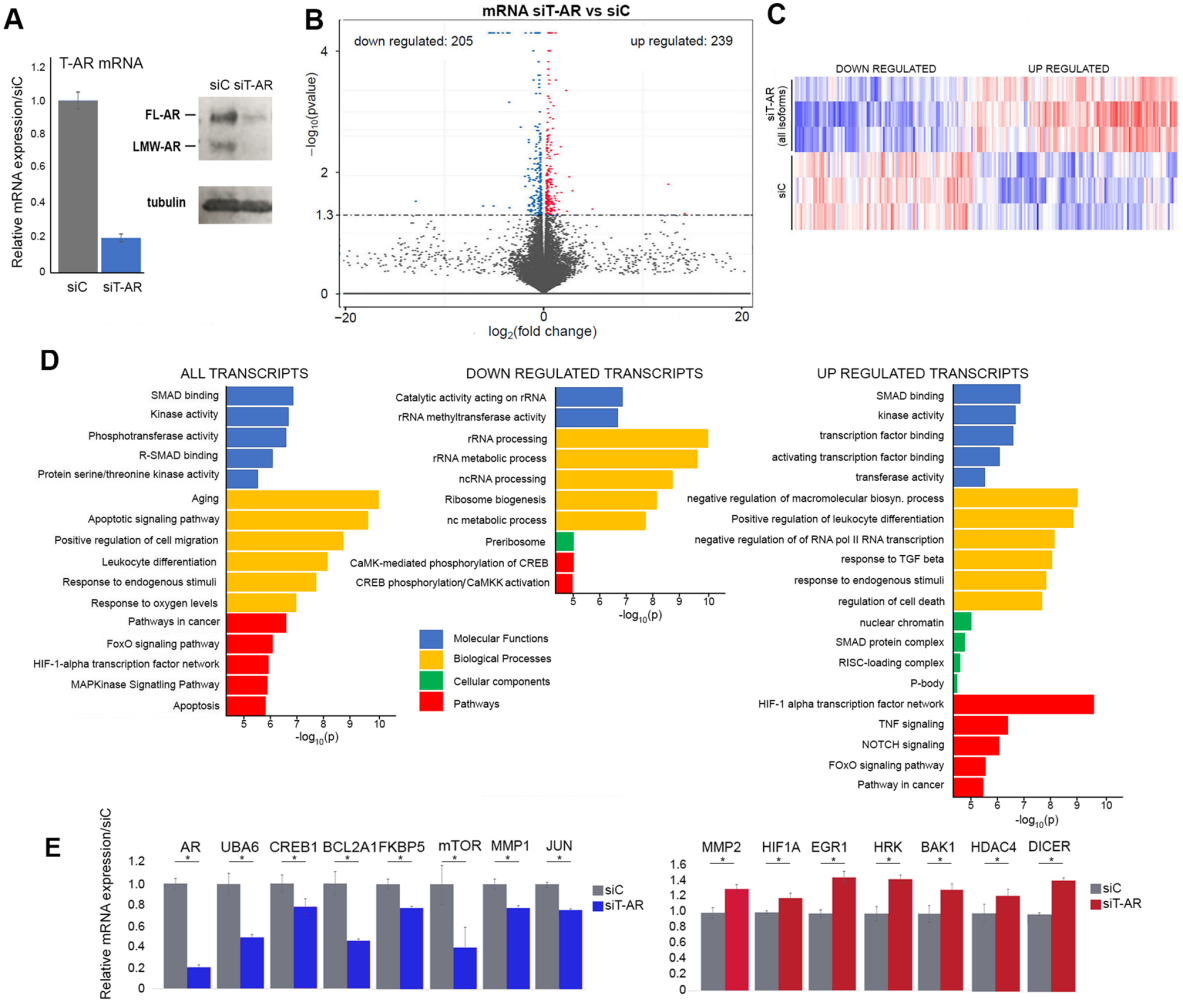
Transcriptome changes following depletion of all AR isoforms in UM-UC-3 cells. (**A**) qRT-PCR (left) and immunoblot analysis (right) validated successful depletion of all AR isoforms following a three-day treatment with AR targeting siRNA. (**B**) Volcano plot analysis indicates similar number of transcripts are repressed (blue) and activated (red) on depletion of all AR isoforms. (**C**) Partial heat map of transcripts repressed and activated on AR depletion when compared to controls. (**D**) Gene enrichment analysis identifies statistically significant molecular function (blue), biological processes (yellow) cellular components (green) and pathways (red) altered on AR depletion. The analysis was conducted on all transcripts (left panel), down regulated transcripts (middle panel) and up regulated transcripts (right panel). (**E**) qRT-PCR verified AR-dependent alterations of selected transcripts. All qRT- PCR analysis was conducted in triplicate. * *P*-value < 0.05. Immunoblots were cropped prior to incubation with specific antibodies.

T-AR depletion was confirmed prior to RNA-seq studies (Fig. 2A). Volcano plot (Fig. 2B) and a representative heatmap (Fig. 2C) indicates that similar numbers of transcripts are elevated (239) and decreased (205) following reduction of AR signaling (Supplementary Dataset File). Gene Ontology (GO) analysis using the ToppGene Suite^18^ and EnrichR^19^ platforms defined molecular functions, biological processes, cellular components, and pathway alterations (Fig. 2D) to obtain a comprehensive understanding of transcript changes. The most altered molecular functions are SMAD binding and kinase activity, while the most altered biological processes are aging, apoptotic signaling, cell migration, response to endogenous stimuli and response to oxygen. The molecular functions, biological processes and cellular components associated with decreased transcripts encode proteins involved in various aspects of RNA metabolism, suggesting a decrease in translational machinery (Fig. 2D middle and right panel). Coordinately, transcripts elevated on AR depletion are associated with a negative regulation of biosynthetic processes, responses to TGFβ and endogenous stimuli, and regulation of apoptosis. Changes in expression of selected transcripts was verified by qRT-PCR (Fig. 2E). Transcription of Mechanistic Target of Rapamycin Kinase (mTOR), Cyclic AMP-Responsive Element-Binding Protein 1 (CREB1), FKBP Prolyl isomerase 5 (FKBP5), Ubiquitin Like Modifier Activating Enzyme 6 (UBA6), Metalloprotease 1 (MMP1), Jun Proto-Oncogene, AP1 Transcription Factor Subunit (JUN), and BCL2 Related Protein A1 (BCL2A1) are decreased, while BCL2 Antagonist/Killer 1 (BAK1), BCL2 Interacting Protein (HRK), Ribonuclease III (DICER), Histone Deacetylase 4 (HDAC4), Early Growth Response 1 (EGR1), and Hypoxia Inducible Factor 1 Subunit Alpha (HIF1A) are increased.

Defining interactions between constituents of five most significantly altered pathways were guided by the Protein String database platform and enhanced by hand annotation (Supplementary Fig. 2). Pathways in cancer includes cell cycle components, mTOR and transcription factors. MAPK pathway includes the CREB1 transcription factor and AP1 transcription factor family (JUN, FOS). Changes in the apoptotic network includes an increase in pro-apoptotic proteins (HRK, BAK) and a decrease in anti-apoptotic BCL2A1, shifting the homeostasis towards apoptosis. The apoptotic pathway is linked to mitochondrial proteins, which are instrumental in oxidative phosphorylation. Notably, multiple HIF1α pathway components are upregulated, indicative of hypoxia.

Long non-coding RNAs (lncRNA) are altered on T-AR depletion where 10 are repressed and 14 are elevated (Supplementary Dataset File). Analysis of the lncRNA targets did not identify significantly altered molecular functions, biological processes, cellular components, or pathways, but this cohort includes several lncRNA previously studied in the cancer context. The repressed small nucleolar host gene 14 (SNHG14), has been reported to promote BlCa cell proliferation and silencing this lncRNA restrains cell viability^22^. Altered deleted in lymphocytic leukemia 2 (DLEU2) expression has been detected in multiple malignancies^23^. Nuclear paraspeckle assembly transcript 1 (NEAT1), scaffold RNA and component of paraspeckles, is elevated. Previous studies reported that in BlCa cells reducing NEAT1 expression augments a reduction in cell proliferation and increases apoptosis on cisplatin treatment^24^, therefore the importance of NEAT in BlCa is unclear.

### FL-AR Isoform regulates multiple histone-encoding transcripts

**Figure 3:**
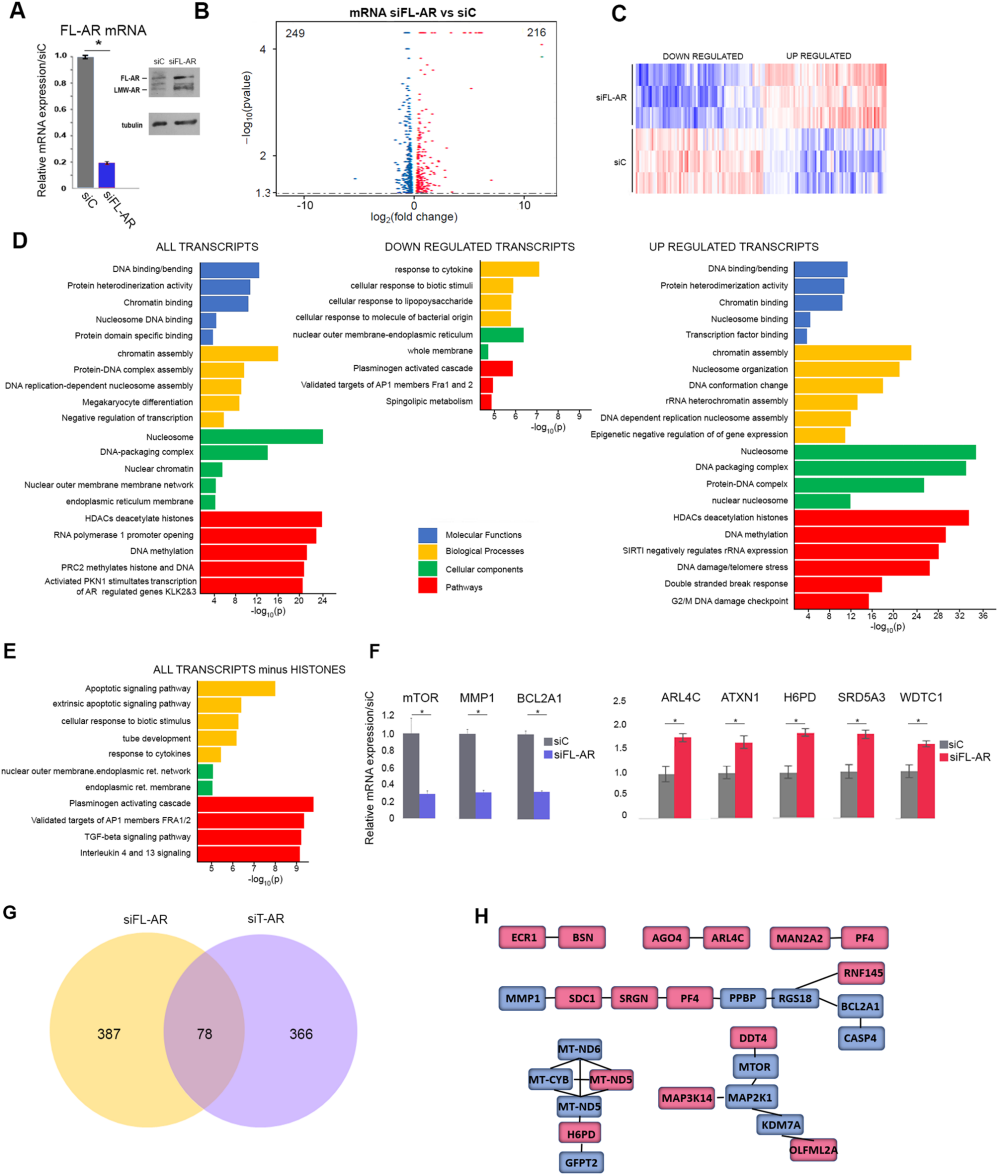
Transcripts regulated by FL-AR. (**A**) qRT-PCR (left) and immunoblot analysis (right) verified a robust depletion of FL-AR mRNA and protein, following a 3-day treatment with FL-AR targeting siRNA. (**B**) Volcano plot of transcripts activated (red) and repressed (blue) on FL-AR depletion. (**C**) Heatmap representation of transcript alteration on FL-AR depletion. (**D**) Gene enrichment analysis shows that histones are highly upregulated on FL-AR depletion. Molecular function (blue), biological processes (yellow) cellular components (green) and pathways (red). (**E**) Re-analysis of transcripts after removing histones identifies additional molecular functions, biological process, cellular components, and pathways altered on FL-AR depletion. (**F**) qRT-PCR verified alterations of selected transcripts. (**G**) Venn diagram of differentially and commonly regulated transcripts on depletion of all AR isoforms (blue) or FL-AR only (yellow). (**H**) Interactions of commonly regulated targets. Downregulated transcripts- blue, upregulated transcripts- red. qRT-PCR studies were in triplicate. * *P*-value < 0.05. Immunoblots were cropped prior to incubation with specific antibodies.

Depletion of FL-AR or treatment with LBD targeting enzalutamide results in a modest reduction of UM-UC-3 cell viability^14,25^. On FL-AR (Fig. 3A) depletion a similar number of transcripts are repressed and elevated (240 and 216 transcripts, respectively, Fig. 3B,C, Supplementary Dataset File). Down regulated transcripts encode proteins involved in biological processes that respond to external stimuli. There is a substantial increase in histone encoding transcripts (Fig. 3D) and accordingly the most altered molecular function, processes, cellular components, and pathways center on chromatin binding, structure, assembly, gene regulation and chromosome repair. Since the large increase in histones could be masking pathways with fewer components, the transcriptome was reanalyzed excluding histones. This revealed significant alteration of plasminogen activating cascade, targets of the AP1 family, TGF-beta, IL4 and IL3 signaling (Fig. 3E,G). Expression of selected transcripts was validated by qRT-PCR and confirmed a decrease of mTOR, MMP1, and BCL2A1, while expression of ADP Ribosylation Factor Like GTPase 4C (ARL4C), Ataxin1 (ATXN1), Hexose-6-Phosphate Dehydrogenase/Glucose 1-Dehydrogenase (H6PD), Steroid 5 Alpha-Reductase 3 (SRD5A3), and WD And Tetratricopeptide Repeats 1 (WDTC1) are elevated (Fig. 3F). The most altered pathways included Plasminogen activated cascade, Fra1 and 2 targets, interleukin 4 and 13 signaling and TGF-β (Supplementary Fig. 3). Comparison of transcripts altered following depletion of T-AR and FL-AR identified 78 commonly regulated transcripts (Fig. 3G) but most transcripts in the two datasets are unique. Three multicomponent interacting sets centered on mitochondrial transcripts, mTOR signaling, and cytokine mediated signaling (Fig. 3H).

### AR-v19 regulated transcriptome includes cell cycle and replication stress associated transcripts

**Figure 4:**
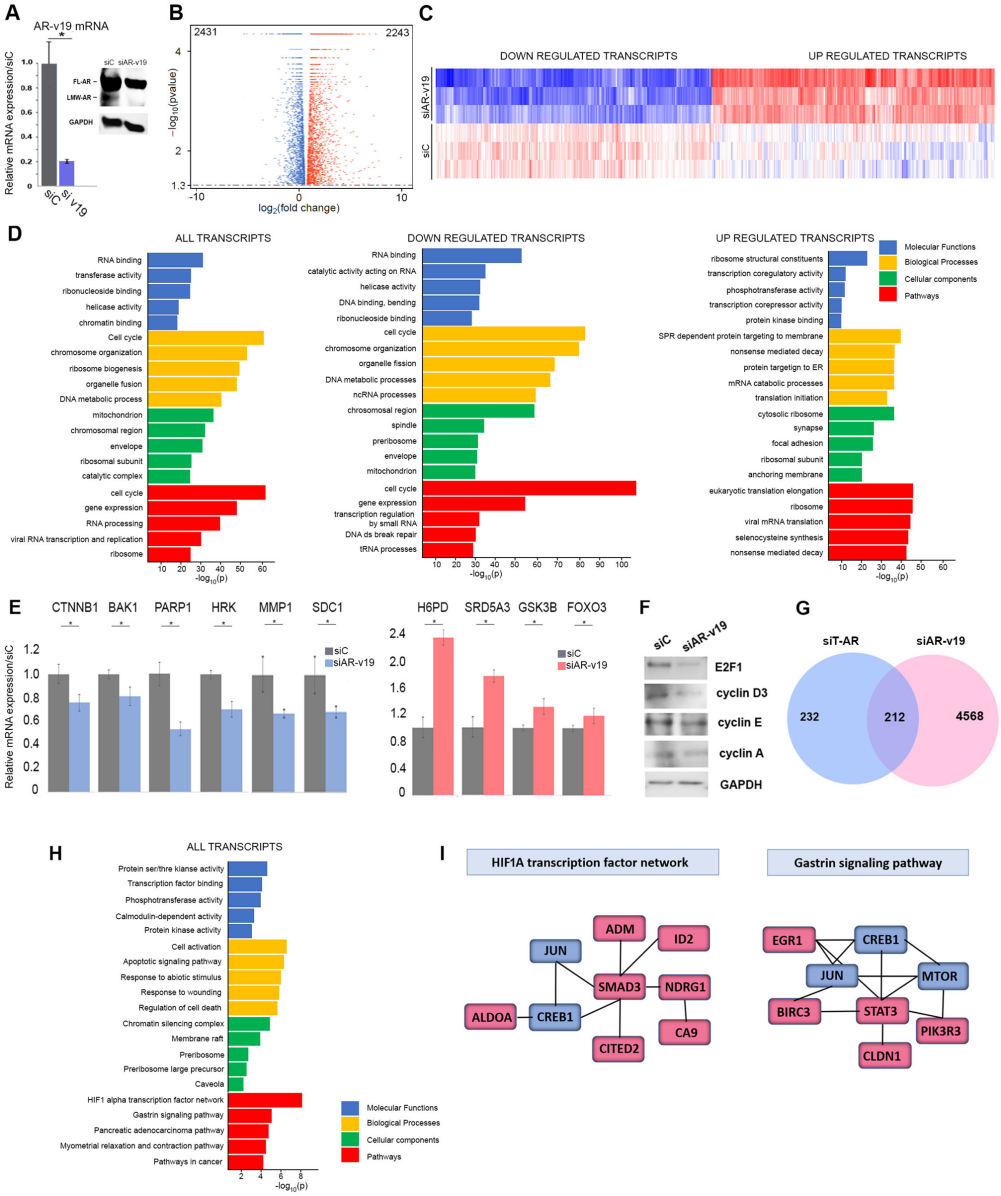
Transcripts regulated on depletion of AR-v19 splice variant. (**A**) qRT-PCR (left panel) and immunoblot (right panel) verification of AR-v19 depletion. (**B**) Volcano plot indicates that on AR-v19 depletion similar numbers of transcripts are activated (red) and repressed (blue). (**C**) Partial heatmap of transcripts altered by AR-v19 depletion. (**D**) Gene enrichment analysis of transcripts altered on AR-v19 shows a steep decline in transcripts involved in all aspect of cell proliferation. (**E**) qRT-PCR verified alterations of selected transcripts. (**F**) Immunoblot analysis of key cell cycle proteins following AR-v19 depletion. (**G**) Venn diagram of differentially and commonly regulated transcripts on depletion of all AR isoforms (blue) or AR-v19 (pink). (**H**) Gene enrichment analysis of transcripts commonly regulated on T-AR and AR-v19 depletion. (**I**) HIF1 and Gastrin Pathways are altered on T-AR and AR-v19 depletion. Downregulated transcripts- blue, upregulated transcripts- red. qRT-PCR analyses were conducted in triplicate. *P-value < 0.05. Immunoblots were cropped prior to incubation with specific antibodies.

AR-v19 depletion resulted in altered regulation of a large number of transcripts where 2243 transcripts are increased and 2431 are decreased (Fig. 4A–C, Supplemental Table 1). The most prominent alterations in molecular function are RNA, ribonucleotide and chromatin binding, transferase and helicase activity resulting in altered cell cycle, ribosomal biogenesis, and DNA metabolic processes while the altered cellular components included mitochondrion, ribosomal subunits, and chromosomal regions. Consistent with these changes, the most significant pathway alterations are cell cycle, gene expression, RNA processing and ribosome (Fig. 4D). qRT-PCR validation confirmed that Poly (ADP-Ribose) Polymerase 1 (PARP1), mTOR, Syndecan 1 (SDC1), Catenin Beta 1 (CTNNB1), BAK1, HRK and MMP1 are decreased, while Glycogen Synthase Kinase 3 Beta (GSK3B), FOXO3, SRD5A3, H6PD are increased (Fig. 4E). Decreased expression of key protein required for cell cycle transversal confirms a decline in proliferation (Fig. 4F). Given the substantial number of altered transcripts, additional analyses focused on the 250 most highly decreased and 250 most highly increased transcripts (Supplementary Dataset File). Overall, the molecular functions, biological processes and cellular components identified in this subset analysis are indicative of a decrease in proliferation and a response to replication stress (Supplementary Dataset File). Comparison of transcripts altered on depletion of T-AR forms and AR-v19 depletion identified 212 transcripts that are commonly regulated (Fig. 4G). GO analysis of this subset identified altered functions, processes, cellular components, and pathways (Fig. 4H) to revealed that the most significantly altered pathways are HIF1A and Gastrin signaling (Fig. 4I).

### Transcript alterations in cells overexpressing AR-v19 are linked to metabolism

**Figure 5:**
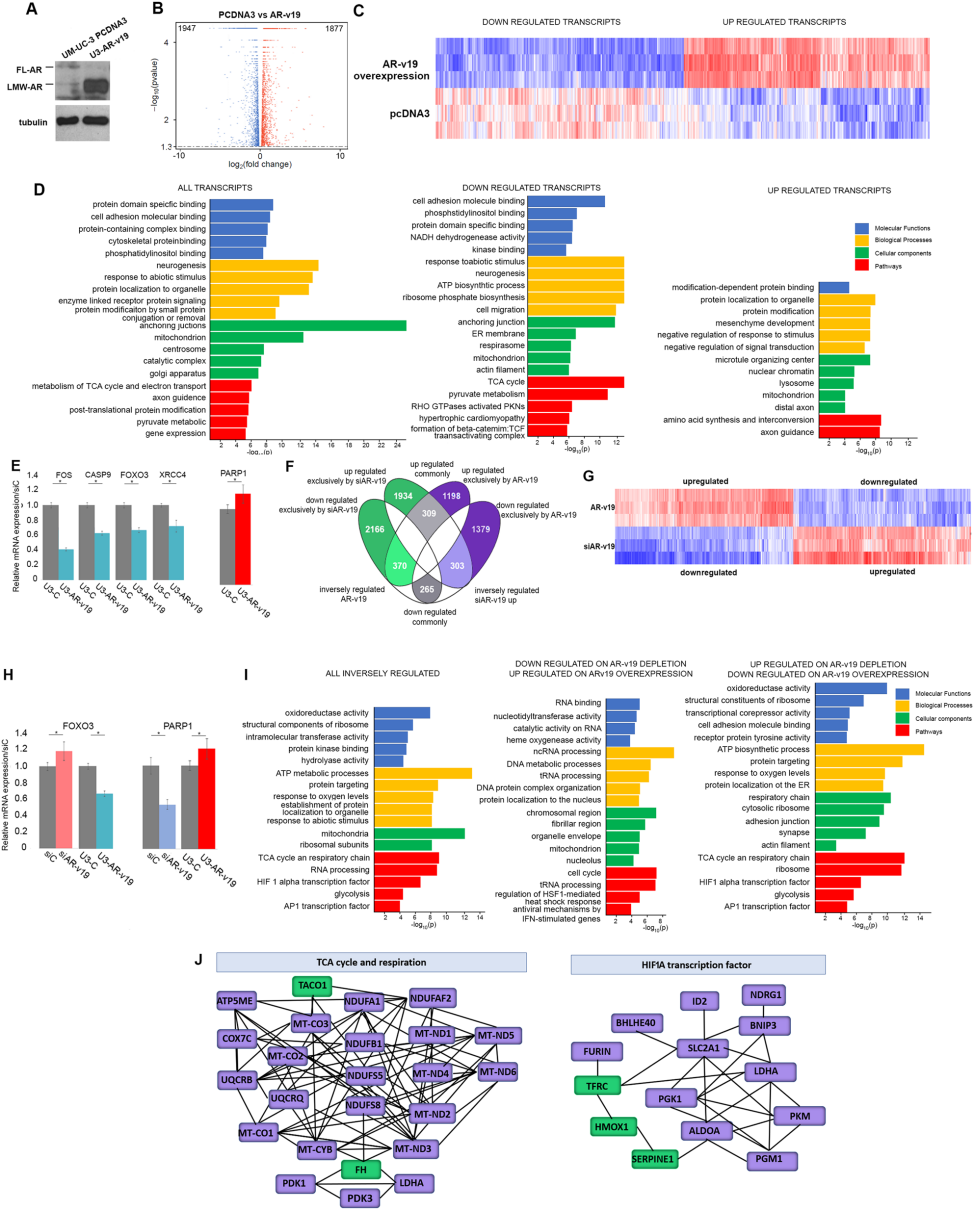
Transcriptome of cells overexpressing the AR-v19 isoform. (**A**) Western blot confirm highly elevated AR-v19 in AR-v19 overexpressing UM-UC-3 cells. (**B**) Volcano plot analysis shows equivalent number of transcripts elevated and decreased in AR-v19 overexpressing cells when compared to UM-UC-3 cells harboring the parental expression vector PCDNA3. (**C**) Heatmap of altered transcripts in AR-v19 overexpressing cells compared to cells harboring the parental vector. (**D**) Molecular function (blue), biological processes (yellow) cellular components (green) and pathways (red) altered in AR-v19 overexpressing cells. (**E**) qRT-PCR validation of transcripts regulated in AR-v19 overexpression. (**F**) Comparison of transcript regulation on AR-v19 depletion and overexpression. Transcripts were divided into those exclusively altered after AR-v19 depletion (green) or AR-v19 overexpression (indigo), and those inversely regulated, upregulated on AR-v19 depletion, and downregulated on AR-v19 overexpression (light green) and downregulated on AR-v19 depletion and upregulated on AR-v19 overexpression (light indigo). Transcripts commonly altered in both models are shown in grey. (**G**) Heatmap of inversely regulated transcripts. (**H**) qRT-PCR validation of transcripts inversely regulated on AR-v19 overexpression or depletion. I Gene enrichment analysis of the identified transcripts. Molecular function (blue), biological processes (yellow) cellular components (green) and pathways (red) inversely altered in AR-v19 depleted and overexpressing cells. (**I**) qRT-PCR validation of selected inversely regulated transcripts. J. Pathway interactions of the most significantly inversely regulated components: up on AR-v19 depletion (indigo), up on AR-v19 overexpression (green). Immunoblots were cropped prior to incubation with specific antibodies.

AR-v19 overexpressing cells (Fig. 5A) were used to identify transcripts that were elevated (1877) or repressed (1947) when compared to cells harboring the parental vector (Fig. 5B,C; Supplementary Dataset File). GO analysis identified protein binding as the prominent altered molecular function (Fig. 5D). Altered biological processes include neurogenesis, response to abiotic stimulus, and protein localization, while the altered cellular components are mitochondrion, centrosome, catalytic complexes and Golgi apparatus. Pathway analysis of decreased transcripts indicated that TCA cycle and pyruvate metabolism as the most altered, while elevated transcripts encode components of amino acid synthesis and axonal guidance pathways. Regulation of selected transcripts was confirmed by qRT-PCR (Fig. 5E). The analysis indicates that increased AR-v19 is altering cellular metabolism.

### Transcripts inversely regulated on AR-v19 depletion and overexpression are associated RNA processing and metabolism

Comparison of AR-v19 k/d and AR-v19 overexpression transcriptomes identified transcripts that were exclusively altered in each data set (AR-v19 depletion-green, AR-v19 overexpression purple), transcripts inversely regulated, upregulated following AR-v19 depletion, downregulated on AR-v19 overexpression (303, light purple) or downregulated on AR-v19 depletion and upregulated on AR-v19 overexpression (370, light green) and transcripts commonly altered in both datasets (grey) (Fig. 5F). A heatmap of inversely regulated transcripts further highlights inversely regulated transcripts (Fig. 5G). Further analysis confirmed that the DNA repair protein PARP1 is decreased on AR-v19 depletion and elevated on AR-v19 overexpression, while the transcription factor FOXO3 is regulated in the inverse manner (Fig. 5H). GO analysis of inversely regulated transcripts found that the most significantly altered molecular functions are oxidoreductase activity and structural component of the ribosome. Significant biological processes are ATP metabolic processes, and the most significant cellular components are mitochondria and ribosomal subunits (Fig. 5I). These alterations culminated in significant changes to TCA cycle, RNA processing, and HIF1α regulated pathway (Fig. 5J).

### Transcripts commonly regulated on depletion of FL-AR, AR-v19 or T- AR include mTOR

**Figure 6:**
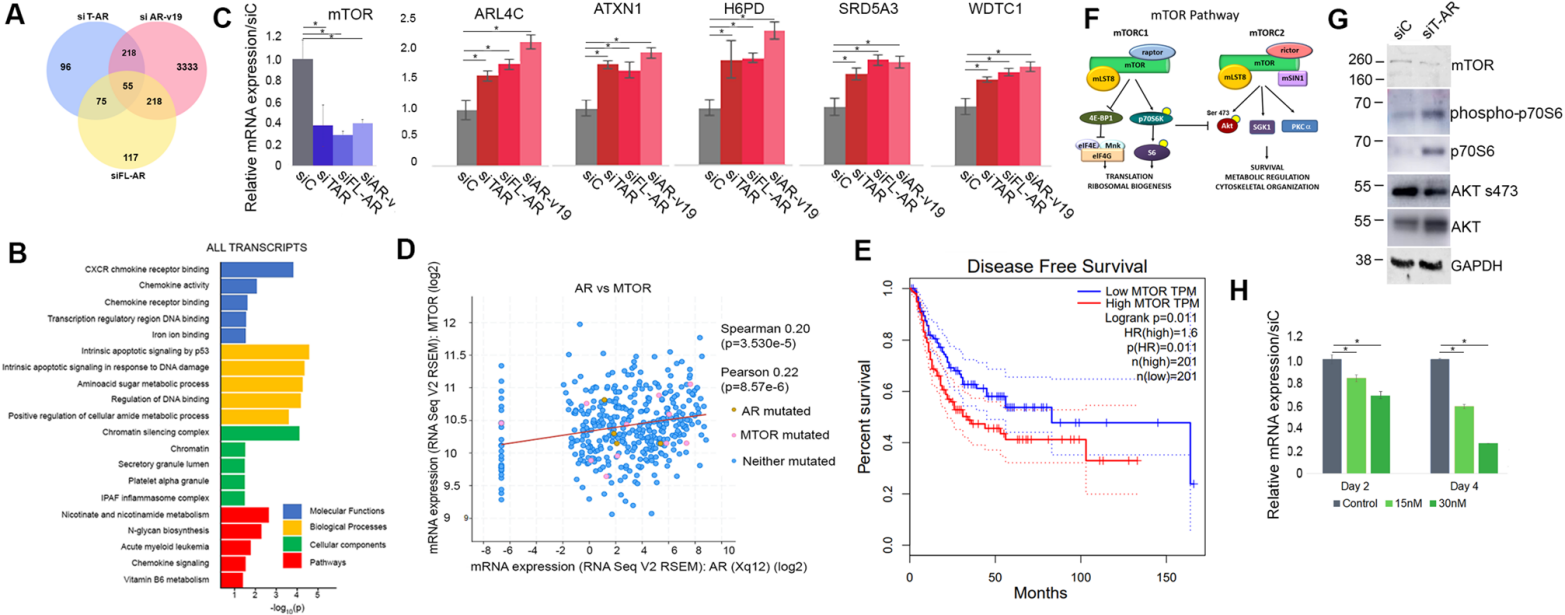
Transcripts commonly regulated on depletion of all or different AR isoforms. (**A**) Venn diagram showing transcripts commonly regulated on depletion of FL-AR, AR-v19 or T-AR (total) isoforms. (**B**) Analysis of commonly regulated molecular function (blue), biological processes (yellow), cellular components (green) and pathways (red). (**C**) Validation of selected transcripts. (**D**) Interrogation of TCGA data identified a statistically significant correlation between AR and mTOR expression in high grade BlCa. (**E**) Kaplan–Meier curves (GEPIA2) identified a significant association between high mTOR levels and decreased disease-free survival. (**F**) Schematic of mTOR complex 1 and 2 signaling pathways. G. Western immunoblot analysis of mTOR and downstream components. (**H**) Pharmacological targeting of both mTOR complexes using INK128 reduces cell viability in a dose and time dependent manner. Immunoblots were cropped prior to incubation with specific antibodies.

Fifty-five transcripts are commonly regulated on depletion of T-AR, FL-AR and AR-v19 (Fig. 6A). The most significantly altered molecular function is CXCR chemokine receptor binding and biological processes significantly deregulated is p53 signaling, apoptotic signaling in response to DNA damage, amino acid sugar metabolism, regulation of DNA binding and regulation of cellular amine metabolic process, while chromatin silencing emerges as the most significantly altered cellular component (Fig. 6B). Significant pathway alterations are not apparent, but the analysis confirmed that AR depletion alters metabolic processes and apoptotic response. Selected commonly regulated transcripts were confirmed by qRT-PCR (Fig. 6C). mTOR is significantly repressed while ARL4C, ATXN1, H6PD, SRD5A and WDTC1 are elevated. An interrogation of primary BlCa tumor transcript data (cBioportal, 413 samples) uncovered a strong statistical correlation between AR and mTOR expression, supporting our results linking AR and mTOR (Fig. 6D). Moreover, elevated mTOR expression significantly correlates with decreased disease-free survival (Fig. 6E).

mTOR drives signaling that is critical for multiple cellular processes including cell growth, survival, metabolism, protein synthesis and autophagy. mTOR is a key component of two complexes, mTORC1 and mTORC2 (Fig. 6F), which initiate two distinct but interrelated signaling cascades which drive translation and ribosomal biogenesis (mTORC1), survival, metabolism and cytoskeletal organization (mTORC2)^26^. Immunoblot analysis confirmed that mTOR protein is reduced on AR depletion (Fig. 6G). Phosphorylation and levels of key mTORC1 and mTORC2 targets were assessed and show that total p70S6 and AKT are elevated. mTORC2 target AKT phosphorylation is reduced, but mTORC1 target p70S6 phosphorylation is increased, indicating that AR depletion has differential effects on the two mTOR complexes (Fig. 6G). Treatment of cells with mTOR targeting sapanisertib results in a dose and time-dependent decrease in cell viability (Fig. 6H).

### Total AR depletion decreases OCR and increases hypoxia signature

Interrogation of cBioportal and GEPIA2^27^ databases was used to determine which AR regulated transcripts significantly correlated with AR expression in primary high-grade BlCa. In addition to mTOR, there is a statistically significant positive correlation between AR and the key transcription factor CREB1, lysine demethylase 7A (KDM7A), caspase 9 (CASP9), syndecn 1 (SDC1), ST3 beta-galactoside alpha-2,3 sialyltransferase 1 (ST3GAL1), catenin Beta 1 (CTNNB1), and (FOXO3) (Supplementary Fig. 4). Statistically significant decreases in disease free survival are evident in individuals where malignancies have elevated expression of CREB1, KDM7A, and forkhead box O-3 (FOXO3), and a decrease in overall survival with higher expression of TNF receptor associated factor 4 (TRAF4) and CASP9, suggesting that these genes may have a role in BlCa tumorigenesis (Supplementary Fig. 5).

**Figure 7:**
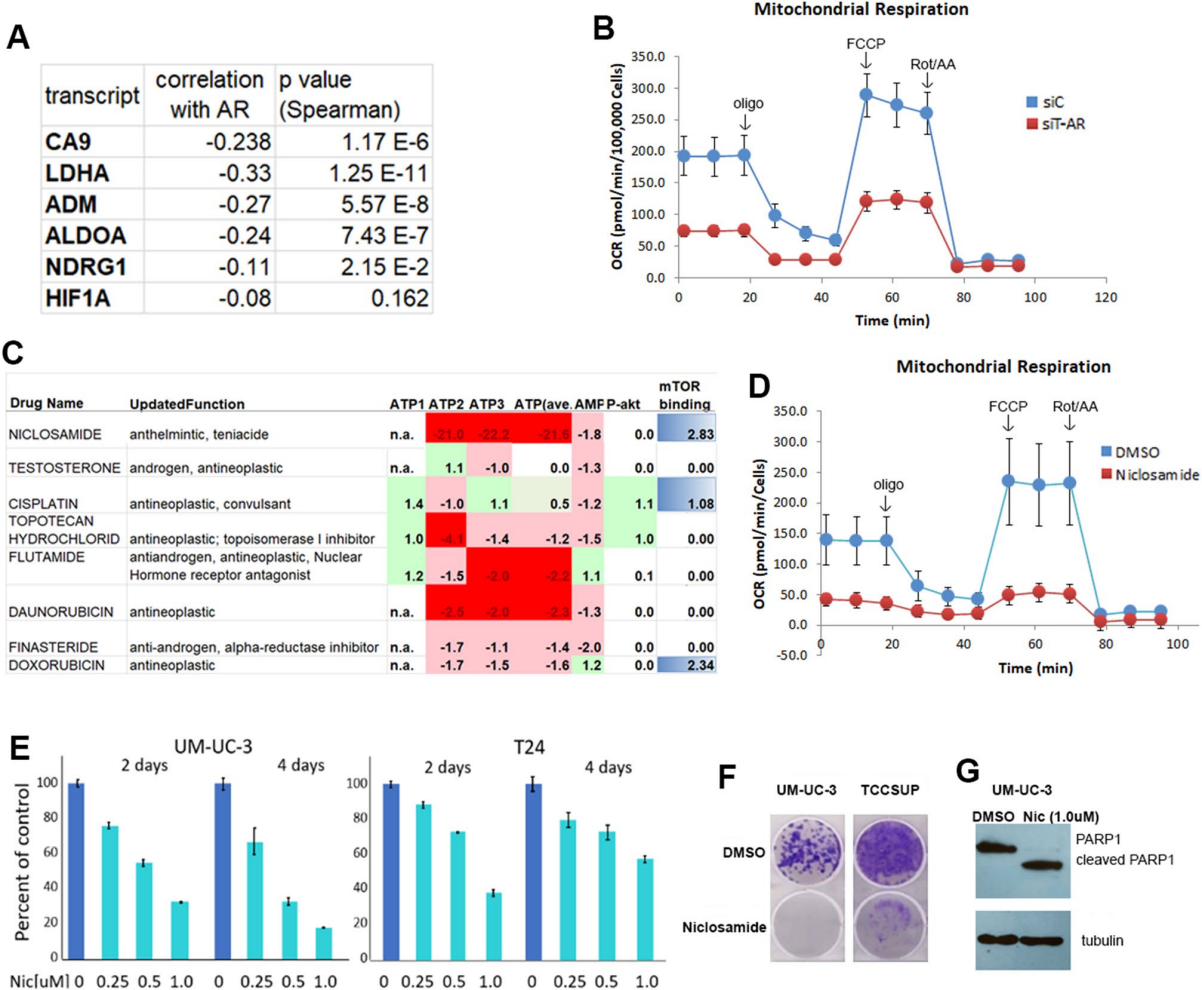
HIF1A components and hypoxia levels inversely correlate with AR expression in high grade BlCa malignancies. (**A**) Interrogation of high grade BlCa malignancies (TCGA 2017) identified statistically significant negative correlations between expression of AR and multiple HIF1A pathway components. (**B**) OCR decrease on total AR depletion is indicative of compromised mitochondrial function. (**C**) An analysis of 1600 agents revealed that niclosamide is a very potent inhibitor of ATPase. The analysis was in triplicate (labeled 1, 2 3) and values were averaged. AMP kinase (AMPK) activity, phospho-ATK (P-AKT) levels and mTOR binding was evaluated. (**D**) OCR decrease on niclosamide treatment is indicative of compromised mitochondrial function. (**E**) Niclosamide treatment decreased cell viability in a time and dose dependent manner in two BlCa cell lines. (**F**) Colony formation assays confirmed the potent effects of niclosamide (0.5uM) following a 10-day treatment in two cellular contexts. G. Complete PARP1 cleavage on niclosamide treatment is indicative of an apoptotic response. Blue- binding. Immunoblots were cropped prior to incubation with specific antibodies.

Significant negative correlations were apparent between AR and HIF1A pathway components carbonic anhydrase 9 (CA9), adrenomedulli (ADM), lactate dehydrogenase A (LDHA), aldolase, fructose-bisphosphate A (ALDOA), and N-myc downstream regulated 1 (NDRG1) (Fig. 7A). The T-AR depletion-dependent increase in hypoxia signature is indicative of decreased oxidative phosphorylation. Analysis of cellular OCR following T-AR depletion uncovered a significant decline in mitochondrial respiration (Fig. 7B). Since AR depletion reduces mitochondrial activity, we analyzed a screen of 1600 clinically used drugs to identify compounds that affect mitochondrial function^28^. The anti-helminth, niclosamide, previously described as a mitochondrial uncoupler in tapeworms and in human cells^29–31^ exhibits very potent ATPase inhibitory activity (Fig. 7C). In addition, in vitro assays found that niclosamide binds mTOR, hence affecting two key identified AR-modulated pathways. As expected niclosamide treated cells have a significant decrease in OCR, consistent with an inhibition of oxidative phosphorylation (Fig. 7D).

### Limiting mitochondrial activity effectively inhibits cell viability and promotes apoptosis

UM-UC-3 cells treated with niclosamide show a dose and time-dependent decline in cell viability (Fig. 7E). Similar effects are observed in T-AR depletion sensitive T24 BlCa cells. Treatment of UM-UC-3 and TCCSUP cells (also sensitive to T-AR depletion) with 0.5 uM niclosamide for 10 days drastically reduces colony formation, confirming drug efficacy (Fig. 7F). Robust PARP1 cleavage indicates that niclosamide induces apoptosis in BlCa cells (Fig. 7G). Overall, these results strongly argue that niclosamide inhibits BlCa viability by inducing apoptosis.

## Discussion

The current study defined BlCa AR-driven transcriptional output to identify exploitable downstream vulnerabilities. We found that T-AR and AR isoforms regulate distinct cohorts of genes, but with an overlapping transcript population, similarly to what has been reported for prostatic malignancies^32–36^. An abrupt cessation of all AR signaling results in the downregulation of transcripts associated with proliferative biosynthetic processes and coordinately, an elevation of transcripts encoding proteins that negatively regulate these processes, pro-apoptotic proteins, and markers of hypoxia arguing that AR is instrumental in promoting metabolic activities.

Our previous studies found that treatment with enzalutamide has a small effect on UM-UC-3 viability suggesting that FL-AR is active in these cells. Notably, the cells are culture in 10% FBS (0.1 nM testosterone)^37,38^ and proliferation is unaltered in steroid depleted media (data not shown), arguing that either very low androgen levels are sufficient for growth, that the cell synthesize sufficient testosterone, or that the AR, but not androgens are essential for cell growth. However, siRNA-mediated depletion of FL-AR retarded cell viability^14^, suggesting that this isoform is active. FL-AR activity is further substantiated by the current study where depletion of the FL-AR elevates expression of multiple histone transcripts. The consequences of an abrupt elevation of multiple histones are not well studied, but in yeast abnormal histone accumulation interferes with chromosome segregation and promotes whole genome duplication^39^, while overexpression of replication-dependent histone genes confers malignancy to dedifferentiated liposarcoma^40^. Restraining FL-AR in the BlCa context may have similar consequences, but this needs to be further explored. The large transcriptome changes following AR-v19 depletion argue that this specific splice variant has a major role in gene expression. Surprisingly, depletion of AR-v19 altered expression of far more transcripts than depletion of T-AR or FL-AR. The reason for this is unclear, but an abrupt depletion of all AR forms in cells that are addicted to this signaling pathway may precipitate an acute stress response that culminates in a rapid induction of programmed cell death. Depletion of a single isoform, while deleterious, may be compensated by the remaining AR, thus slowing the decline in cell viability. Comparison of transcription alterations on AR-v19 k/d and overexpression reiterated that depletion and overexpression of a transcription factor does not drive gene expression in an inverse manner.

There is a modest overlap in transcripts commonly regulated on depletion of T-AR, FL-AR and AR-v19. Several elevated transcripts have previously been associated with malignancies. WDTC1 has oncogenic properties in colorectal cancer^41^. ATXN1 regulates epithelial mesenchymal transition (EMT) in cervical carcinoma cells where its depletion promotes migration and invasion^42^. ARL4C, a target of Wnt/β-catenin and Ras signaling, positively correlates with EMT on AR depletion in PCa^43^. H6PD is elevated in prostatic malignancies treated with AR-limiting enzalutamide^44^, and SRD5A3 is elevated in PCa when AR signaling is restrained^44^. SRD5A3 converts testosterone to the more potent dihydrotestosterone^45^ thus SRD5A3 upregulation on AR depletion suggests that a decline of AR signaling triggers a compensatory increase in SRD5A3 to restore signaling. While certain transcripts are similarly regulated in BlCa and PCa, there are notable differences. Myc, highly AR-regulated in PCa^12^, is not AR regulated in BlCa. KLK2 and 3 are not expressed while TMPRSS2 is expressed at very low levels and not AR regulated, hence some well described AR targets in PCa, are not AR-regulated in BlCa.

A previous study used a different approach where a UM-UC-3 subclone with highly elevated AR expression when compared to UM-UC-3 parent cells, was stimulated with 10 nmol/L R1881 and subjected to CHiP-seq studies to identify AR binding regions^46^. RNA from identically treated cells was used in expression microarray studies leading to the identification of 96 direct AR target genes. A comparison of the identified direct AR target genes with transcripts altered by depletion of T-AR, FL-AR or AR-v19 found that of the 51 of the 96 were regulated by on T-AR, FL-AR or AV-v19 depletion. This included FKBP5 and CD44 (Supplementary Fig. 6). However, most of the identified transcripts were not inversely regulated on AR depletion vs R1881-dependent AR activation, suggesting the two cell lines while similar has distinct features or additional factors have a substantial role in regulating AR-dependent gene expression.

mTOR is decreased on depletion of T-AR, FL-AR and AR-v19 and the AR/mTOR correlation expends to high grade malignancies, arguing that this linkage is a common feature of BlCa. The importance of AR in driving mTOR is documented in the context of muscle cells. In castrated rodents, testosterone supplementation improved muscle and bone strength by mTOR activation^47^ where it optimizes muscle protein levels by regulating the balance of mTOR and AMPK^48^. In PCa, elevated or deregulated mTOR signaling is a major cause of castration resistance, hence targeting this pathway is an attractive strategy^49^. Interaction between AR and mTOR includes direct binding of nuclear mTOR to AR to affect gene transcription^50^. In PCa cells limiting AR signaling promotes an increase of mTORC1 downstream target p70S6 phosphorylation, but not the mTORC2 target AKT. Moreover, C1 and C2 regulate each other, where depletion of the critical C1 protein raptor enhances C2 signaling, while depletion of the C2 protein rictor elevated C1 signaling^51^. In BlCa AR depletion also increases phospho p70S6 levels, therefore there are similarities between AR mTOR regulation between PCa and BlCa. However, thus far, clinical trials limiting mTOR activity have shown limited efficacy^52^, but studies using novel mTOR targeting agents or a combination of agents are ongoing and may identify formulations that can effectively limit BlCa tumorigenesis.

Increased expression of HIF1A network components on AR depletion was reinforced by analysis of high grade BlCa. Multiple transcripts indicative of hypoxia strongly negatively correlate with AR. An elevation of a hypoxic signature and reduction of OCR on T-AR depletion is indicative of compromised mitochondrial activity and suggested a targetable vulnerability. AR interplay with mitochondria is complex. AR can directly regulate mitochondrial gene expression, where in muscle C2C12 cells AR promotes mitochondrial gene transcription driving mitochondrial biogenesis^53^, while in PCa cells mitochondrial AR serves to reduce mitochondrial function by regulating OXPHOS enzymatic activity^54^. In castration resistant PCa, MPC2 is a direct AR target and its inhibition restricted OCR^55^. Our analysis did not identify MPC2 as an AR regulated transcript, but AR inhibition has a similar effect on OCR, suggesting that oxidative phosphorylation may be regulated by alternative mechanisms in BlCa, but the overall outcome is similar.

Several feature of the antihelminth niclosamide make it particularly attractive drug for mimicking the effect of AR depletion. (1) Niclosamide has a robust effect on ATPase and treatment of BlCa cells reduce OCR. (2) Niclosamide exhibits mTOR binding. (3) In preclinical and a limited number of clinical trials niclosamide or novel niclosamide formulations exhibit an acceptable toxicity profile^56,57^. Niclosamide was developed to target parasites and early studies reported its effect on uncoupling oxidative phosphorylation^58^. Subsequent studies described additional niclosamide targets and it is unclear if there are multiple niclosamide targets or if there is substantial crosstalk between affected pathways. Niclosamide affects mTOR activity and is a potent inhibitor of Wnt/β-catenin and STAT3 signaling^59,60^. In PCa, niclosamide inhibits AR splice variants to overcome enzalutamide resistance^61^, but we did not observe this effect in BlCa cells, suggesting that niclosamide’s effects on AR are cell context specific. However, niclosamide’s suitability is hindered by its poor solubility and brief plasma half-life^62^. Encouragingly, newer formulations are overcoming these obstacles, repurposing this drug as a cancer therapeutic that may be utilized for the treatment of high grade BlCa^56,57^.

In conclusion, the BlCa studies herein identified T-AR and isoform dependent transcriptomes and uncovered molecular processes and pathways associated with the regulated transcriptomes. Cross referencing with AR regulated transcribes using the cBioportal database validated transcripts that are positively and negatively associated with AR expression. mTOR positively correlated with AR expression, mTOR signaling is deregulated on AR-depletion and pharmachological targeting of mTOR resulted in a significant decline in cell viability. Conversely transcripts that are indicative of hypoxia are negatively associated with AR expression, arguing for an AR effect on mitochondrial activity. Depletion of AR resulted in a sharp decline in OCR and targeting mitochondrial activity with the potent ATPase inhibitor niclosamide decreased OCR and reduced cell viability in a dose and time dependent manner. Taken together these studies found that in BlCa the AR drives metabolism and revealed that compromising mitochondrial activity is a targetable vulnerability.

## Supplementary Information

Supplementary Information.

## Data Availability

All data generated or analyzed during this study are included in this published article (Supplementary Dataset File). The datasets generated during the current study are available under accession number GEO Accession GSE208018: https://www.ncbi.nlm.nih.gov/geo/query/acc.cgi?acc=GSE208018.

## References

[1] Siegel RL, Miller KD, Fuchs HE, Jemal A (2021). Cancer Statistics, 2021. CA Cancer J. Clin..

[2] Knowles MA, Hurst CD (2015). Molecular biology of bladder cancer: new insights into pathogenesis and clinical diversity. Nat. Rev. Cancer.

[3] Zhu C, Boutros PC (2021). Sex differences in cancer genomes: Much learned, more unknown. Endocrinology.

[4] Ide H, Miyamoto H (2021). Sex hormone receptor signaling in bladder cancer: A potential target for enhancing the efficacy of conventional non-surgical therapy. Cells.

[5] Viswambaram P, Hayne D (2020). Gender discrepancies in bladder cancer: potential explanations. Expert. Rev. Anticancer Ther..

[6] Mancini M, Righetto M, Baggio G (2020). Spotlight on gender-specific disparities in bladder cancer. Urologia.

[7] Laor E, Schiffman ZJ, Braunstein JD, Reid RE, Tolia BM, Koss LG (1985). Androgen receptors in bladder tumors. Urology.

[8] Mizushima T, Tirador KA, Miyamoto H (2017). Androgen receptor activation: A prospective therapeutic target for bladder cancer?. Expert. Opin. Ther. Targets.

[9] Johnson DT, Hooker E, Luong R, Yu EJ, He Y, Gonzalgo ML (2016). Conditional expression of the androgen receptor increases susceptibility of bladder cancer in mice. PLoS ONE.

[10] Wang CS, Li CC, Juan YS, Wu WJ, Lee HY (2020). 5alpha-reductase inhibitors impact prognosis of urothelial carcinoma. BMC Cancer.

[11] Stortz M, Presman DM, Pecci A, Levi V (2021). Phasing the intranuclear organization of steroid hormone receptors. Biochem. J..

[12] Isaacs JT (2018). Resolving the Coffey Paradox: What does the androgen receptor do in normal vs. malignant prostate epithelial cells?. Am. J. Clin. Exp. Urol..

[13] Messner EA, Steele TM, Tsamouri MM, Hejazi N, Gao AC, Mudryj M (2020). The androgen receptor in prostate cancer: Effect of structure, ligands and spliced variants on therapy. Biomedicines.

[14] Katleba K, Lombard AP, Tsamouri MM, Baek HB, Nishida KS, Libertini SJ (2021). Depletion of androgen receptor low molecular weight isoform reduces bladder tumor cell viability and induces apoptosis. Cancer Lett..

[15] Hu R, Dunn TA, Wei S, Isharwal S, Veltri RW, Humphreys E (2009). Ligand-independent androgen receptor variants derived from splicing of cryptic exons signify hormone-refractory prostate cancer. Cancer Res..

[16] Kim D, Pertea G, Trapnell C, Pimentel H, Kelley R, Salzberg SL (2013). TopHat2: Accurate alignment of transcriptomes in the presence of insertions, deletions and gene fusions. Genome Biol..

[17] Trapnell C, Roberts A, Goff L, Pertea G, Kim D, Kelley DR (2012). Differential gene and transcript expression analysis of RNA-seq experiments with TopHat and Cufflinks. Nat. Protoc..

[18] Chen J, Bardes EE, Aronow BJ, Jegga AG (2009). ToppGene Suite for gene list enrichment analysis and candidate gene prioritization. Nucl. Acids Res..

[19] Chen EY, Tan CM, Kou Y, Duan Q, Wang Z, Meirelles GV (2013). Enrichr: Interactive and collaborative HTML5 gene list enrichment analysis tool. BMC Bioinf..

[20] Robertson AG, Kim J, Al-Ahmadie H, Bellmunt J, Guo G, Cherniack AD (2017). Comprehensive molecular characterization of muscle-invasive bladder cancer. Cell.

[21] Cerami E, Gao J, Dogrusoz U, Gross BE, Sumer SO, Aksoy BA (2012). The cBio cancer genomics portal: An open platform for exploring multidimensional cancer genomics data. Cancer Discov..

[22] Feng R, Li Z, Wang X, Ge G, Jia Y, Wu D (2021). Silenced lncRNA SNHG14 restrains the biological behaviors of bladder cancer cells via regulating microRNA-211-3p/ESM1 axis. Cancer Cell Int..

[23] Ghafouri-Fard S, Dashti S, Farsi M, Taheri M (2021). Deleted in lymphocytic leukemia 2 (DLEU2): An lncRNA with dissimilar roles in different cancers. Biomed. Pharmacother..

[24] Zhao W, Li W, Jin X, Niu T, Cao Y, Zhou P (2019). Silencing long non-coding RNA NEAT1 enhances the suppression of cell growth, invasion, and apoptosis of bladder cancer cells under cisplatin chemotherapy. Int. J. Clin. Exp. Pathol..

[25] Kawahara T, Ide H, Kashiwagi E, El-Shishtawy KA, Li Y, Reis LO (2016). Enzalutamide inhibits androgen receptor-positive bladder cancer cell growth. Urol. Oncol..

[26] Magaway C, Kim E, Jacinto E (2019). Targeting mTOR and metabolism in cancer: Lessons and innovations. Cells.

[27] Tang Z, Kang B, Li C, Chen T, Zhang Z (2019). GEPIA2: An enhanced web server for large-scale expression profiling and interactive analysis. Nucleic Acids Res..

[28] Datta S, Tomilov A, Cortopassi G (2016). Identification of small molecules that improve ATP synthesis defects conferred by Leber's hereditary optic neuropathy mutations. Mitochondrion.

[29] Pampori NA, Singh G, Srivastava VM (1984). Energy metabolism in Cotugnia digonopora and the effect of anthelmintics. Mol. Biochem. Parasitol.

[30] MacDonald ML, Lamerdin J, Owens S, Keon BH, Bilter GK, Shang Z (2006). Identifying off-target effects and hidden phenotypes of drugs in human cells. Nat. Chem. Biol..

[31] Li Y, Li PK, Roberts MJ, Arend RC, Samant RS, Buchsbaum DJ (2014). Multi-targeted therapy of cancer by niclosamide: A new application for an old drug. Cancer Lett..

[32] Hu R, Lu C, Mostaghel EA, Yegnasubramanian S, Gurel M, Tannahill C (2012). Distinct transcriptional programs mediated by the ligand-dependent full-length androgen receptor and its splice variants in castration-resistant prostate cancer. Cancer Res..

[33] Basil P, Robertson MJ, Bingman WE, Dash AK, Krause WC, Shafi AA (2022). Cistrome and transcriptome analysis identifies unique androgen receptor (AR) and AR-V7 splice variant chromatin binding and transcriptional activities. Sci. Rep..

[34] Liang J, Wang L, Poluben L, Nouri M, Arai S, Xie L (2021). Androgen receptor splice variant 7 functions independently of the full length receptor in prostate cancer cells. Cancer Lett..

[35] Kounatidou E, Nakjang S, McCracken SRC, Dehm SM, Robson CN, Jones D (2019). A novel CRISPR-engineered prostate cancer cell line defines the AR-V transcriptome and identifies PARP inhibitor sensitivities. Nucl. Acids Res..

[36] He Y, Lu J, Ye Z, Hao S, Wang L, Kohli M (2018). Androgen receptor splice variants bind to constitutively open chromatin and promote abiraterone-resistant growth of prostate cancer. Nucl. Acids Res..

[37] Sedelaar JP, Isaacs JT (2009). Tissue culture media supplemented with 10% fetal calf serum contains a castrate level of testosterone. Prostate.

[38] Song W, Khera M (2014). Physiological normal levels of androgen inhibit proliferation of prostate cancer cells in vitro. Asian J. Androl..

[39] Miles DM, Desdouets C, Geli V (2019). Histone stress: An unexplored source of chromosomal instability in cancer?. Curr. Genet..

[40] Yoo Y, Park SY, Jo EB, Choi M, Lee KW, Hong D (2021). Overexpression of replication-dependent histone signifies a subset of dedifferentiated liposarcoma with increased aggressiveness. Cancers (Basel).

[41] Wang X, Cai Q, Ping J, Diaz-Zabala H, Xia Y, Guo X (2022). The putative oncogenic role of WDTC1 in colorectal cancer. Carcinogenesis.

[42] Kang AR, An HT, Ko J, Kang S (2017). Ataxin-1 regulates epithelial-mesenchymal transition of cervical cancer cells. Oncotarget.

[43] Hu Q, Masuda T, Sato K, Tobo T, Nambara S, Kidogami S (2018). Identification of ARL4C as a peritoneal dissemination-associated gene and its clinical significance in gastric cancer. Ann. Surg. Oncol..

[44] Li J, Berk M, Alyamani M, Sabharwal N, Goins C, Alvarado J (2021). Hexose-6-phosphate dehydrogenase blockade reverses prostate cancer drug resistance in xenograft models by glucocorticoid inactivation. Sci. Transl. Med..

[45] Uemura M, Tamura K, Chung S, Honma S, Okuyama A, Nakamura Y (2008). Novel 5 alpha-steroid reductase (SRD5A3, type-3) is overexpressed in hormone-refractory prostate cancer. Cancer Sci..

[46] Sottnik JL, Vanderlinden L, Joshi M, Chauca-Diaz A, Owens C, Hansel DE (2021). Androgen receptor regulates CD44 expression in bladder cancer. Cancer Res..

[47] Hourde C, Jagerschmidt C, Clement-Lacroix P, Vignaud A, Ammann P, Butler-Browne GS (2009). Androgen replacement therapy improves function in male rat muscles independently of hypertrophy and activation of the Akt/mTOR pathway. Acta Physiol. (Oxf.).

[48] Serra C, Sandor NL, Jang H, Lee D, Toraldo G, Guarneri T (2013). The effects of testosterone deprivation and supplementation on proteasomal and autophagy activity in the skeletal muscle of the male mouse: differential effects on high-androgen responder and low-androgen responder muscle groups. Endocrinology.

[49] Pisano C, Tucci M, Di Stefano RF, Turco F, Scagliotti GV, Di Maio M (2021). Interactions between androgen receptor signaling and other molecular pathways in prostate cancer progression: Current and future clinical implications. Crit. Rev. Oncol. Hematol..

[50] Giguere V (2020). DNA-PK, nuclear mTOR, and the androgen pathway in prostate cancer. Trends Cancer.

[51] Wang Y, Mikhailova M, Bose S, Pan CX, deVere White RW, Ghosh PM (2008). Regulation of androgen receptor transcriptional activity by rapamycin in prostate cancer cell proliferation and survival. Oncogene.

[52] Huan J, Grivas P, Birch J, Hansel DE (2022). Emerging roles for mammalian target of rapamycin (mTOR) complexes in bladder cancer progression and therapy. Cancers (Basel).

[53] Pronsato L, Milanesi L, Vasconsuelo A (2020). Testosterone induces up-regulation of mitochondrial gene expression in murine C2C12 skeletal muscle cells accompanied by an increase of nuclear respiratory factor-1 and its downstream effectors. Mol. Cell Endocrinol..

[54] Bajpai P, Koc E, Sonpavde G, Singh R, Singh KK (2019). Mitochondrial localization, import, and mitochondrial function of the androgen receptor. J. Biol. Chem..

[55] Bader DA, Hartig SM, Putluri V, Foley C, Hamilton MP, Smith EA (2019). Mitochondrial pyruvate import is a metabolic vulnerability in androgen receptor-driven prostate cancer. Nat. Metab..

[56] Parikh M, Liu C, Wu CY, Evans CP, Dall'Era M, Robles D (2021). Phase Ib trial of reformulated niclosamide with abiraterone/prednisone in men with castration-resistant prostate cancer. Sci. Rep..

[57] Reddy GB, Kerr DL, Spasojevic I, Tovmasyan A, Hsu DS, Brigman BE (2020). Preclinical testing of a novel niclosamide stearate prodrug therapeutic (NSPT) shows efficacy against osteosarcoma. Mol. Cancer Ther..

[58] Weinbach EC, Garbus J (1969). Mechanism of action of reagents that uncouple oxidative phosphorylation. Nature.

[59] Fonseca BD, Diering GH, Bidinosti MA, Dalal K, Alain T, Balgi AD (2012). Structure-activity analysis of niclosamide reveals potential role for cytoplasmic pH in control of mammalian target of rapamycin complex 1 (mTORC1) signaling. J. Biol. Chem..

[60] Arend RC, Londono-Joshi AI, Gangrade A, Katre AA, Kurpad C, Li Y (2016). Niclosamide and its analogs are potent inhibitors of Wnt/beta-catenin, mTOR and STAT3 signaling in ovarian cancer. Oncotarget.

[61] Liu C, Lou W, Zhu Y, Nadiminty N, Schwartz CT, Evans CP (2014). Niclosamide inhibits androgen receptor variants expression and overcomes enzalutamide resistance in castration-resistant prostate cancer. Clin. Cancer Res..

[62] Yang W, de Villiers MM (2005). Effect of 4-sulphonato-calix[n]arenes and cyclodextrins on the solubilization of niclosamide, a poorly water soluble anthelmintic. AAPS J..

