# Supplementary material for: Androgen receptor-dependent regulation of metabolism in high grade bladder cancer cells
Source: Sci Rep. 2023 Jan 31;13:1762. doi: 10.1038/s41598-023-28692-z (PMC9889754; doi:10.1038/s41598-023-28692-z)
Supplement: Supplementary file 1 — Supplementary Information. [file 41598_2023_28692_MOESM1_ESM.pdf]

Androgen receptor-dependent regulation of metabolism in high grade bladder cancer cells

Kimberley D. Katleba, Maria-Malvina Tsamouri, Maitreyee Jathal, Han Bit Baek, Rebecca B. Armenta, Clifford G. Tepper, Gino Cortopassi, Paramita M. Ghosh, Maria Mudryj

Supplementary Figure 1. qRT-PCR primers used in current study.

Primers	Forward	Reverse	Size (bp)
TAR	TGGATAGCTACTCCGGACCTT	CCCAGAAGCTTCATCTCCAC	125
AR-LBD	GCCTTGCTCTCTAGCCTCAA	GTCGTCCACGTGTAAGTTGC	102
AR-v19	TGAAGCAGGGATGACTCTGG	CCAAATCGAAGTGGATTAATGA	103
β-Actin	GGAAATCGTGCGTGACATTAAG	AGCTCGTAGCTCTTCTCCA	108
UBA6	AGAGGTACGTTCTTGGAGACAC	TGGTTCCTAGATCCCATGCTT	171
CREB1	ATTCACAGGAGTCAGTGGATAGT	CACCGTTACAGTGGTGATGG	182
BCL2A1	TACAGGCTGGCTCAGGACTAT	CGCAACATTTTGTAGCACTCTG	96
FKBP5	GACCGCAGGATATACGCCAACAT	AGTCTTCTTGCCCATTGCTTTATTG	87
mTOR	GCAGATTTGCCAACTATCTTCGG	CAGCGGTAAAAGTGTCCCCTG	114
MMP1	CTCTGGAGTAATGTCACACCTCT	TGTTGGTCCACCTTTCATCTTC	199
JUN	GGCTGGTGTTTCGGGAGTGT	CGCCGCCTTCTGGTCTTTAC	261
MMP2	AGCGAGTGGATGCCGCCTTTAA	CATTCCAGGCATCTGCGATGAG	138
HIF1A	CACCACAGGACAGTACAGGAT	CGTGCTGAATAATACCACTCACA	146
EGR1	CTTCAACCCTCAGGCGGACA	GGAAAAGCGGCCAGTATAGGT	160
HRK	GGCAGGCGGAACTTGTAGGAAC	TCCAGGCGCTGTCTTTACTCTCC	197
BAK1	GTTTTCCGCAGCTACGTTTTT	GCAGAGGTAAGGTGACCATCTC	97
HDAC4	CCTGGGAATGTACGACGCC	CCCGTCTTTCCTGCGTAAC	136
DICER	TGCTATGTCGCCTTGAATGTT	AATTTCTCGATAGGGGTGGTCTA	114
ARL4C	CTGCTGGTCATCGCCAACA	ATAGGTGGTGGCCGGGATAA	102
ATXN1	TCGTCATGCAATACGCCGAC	TACGGGTGAGGAACCGACT	202
H6PD	GCAGAGCACAAGGATCAGTTC	GGCAGCTACTGTTGATGTTGC	196
SRD5A3	TCTGCCCATCAGTATAAGTGCC	GTGGAACCCAAAGGTGACGG	180
WDTC1	ACACTCGGAGGTGCTGATTG	GCGGATGTCATAGAGCCTCAC	139
CTNNB1	CATCTACACAGTTTGATGCTGCT	GCAGTTTTGTCAGTTCAGGGA	150
PARP1	CGGAGTCTTCGGATAAGCTCT	TTTCCATCAAACATGGGCGAC	136
SDC1	CCACCATGAGACCTCAACCC	GCCACTACAGCCGTATTCTCC	192
GSK3B	AGACGCTCCCTGTGATTTATGT	CCGATGGCAGATTCCAAAGG	89
FOXO3	TCACGCACCAATTCTAACGC	CACGGCTTGCTTACTGAAGG	155
FOS	GGGGCAAGGTGGAACAGTTAT	CCGCTTGGAGTGTATCAGTCA	126
CASP9	CTCAGACCAGAGATTCGCAAAC	GCATTTCCCCTCAAACTCTCAA	116
XRCC4	ATGTTGGTGAACTGAGAAAAGCA	GCAATGGTGTCCAAGCAATAAC	208

Supplementary Figure 2. Identified pathway component interactions of five top pathways on T-AR depletion. Downregulated transcripts- blue, upregulated transcripts- red.

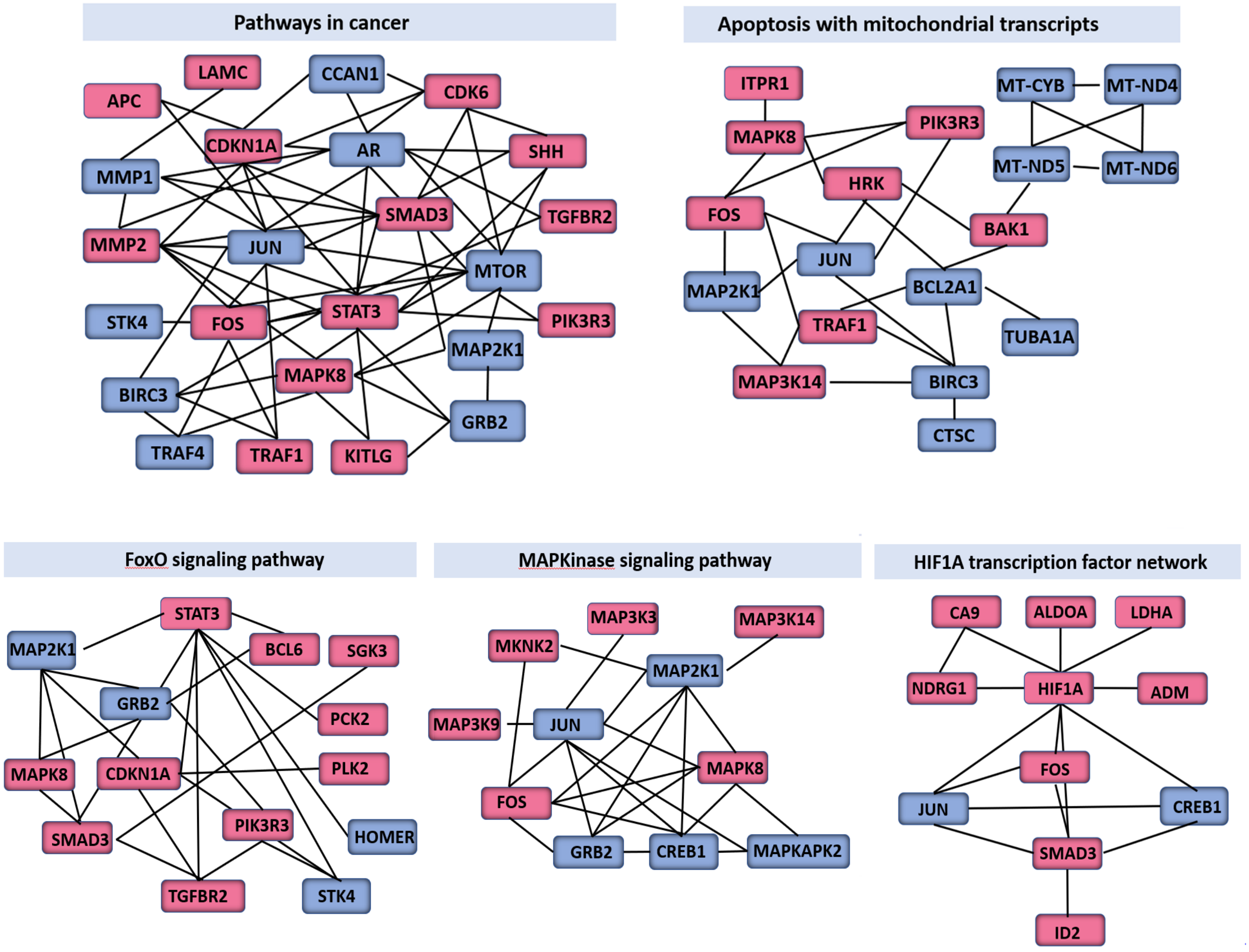

Supplementary Figure 3. Pathway component interactions of pathways most significantly altered on FL-AR depletion. Downregulated transcripts- blue, upregulated transcripts- red.

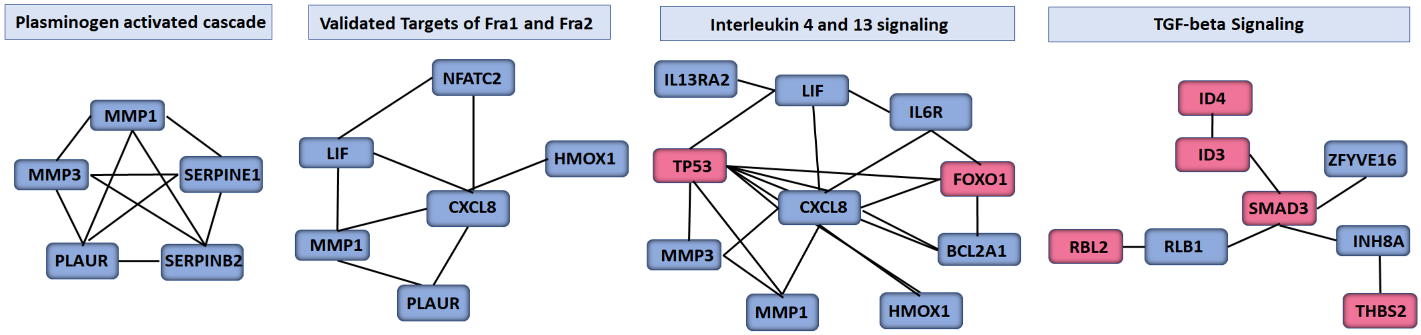

Supplementary Figure 4. TCGA (cBioportal) analysis identified positive and negative correlations between AR and identified AR-regulated transcript.

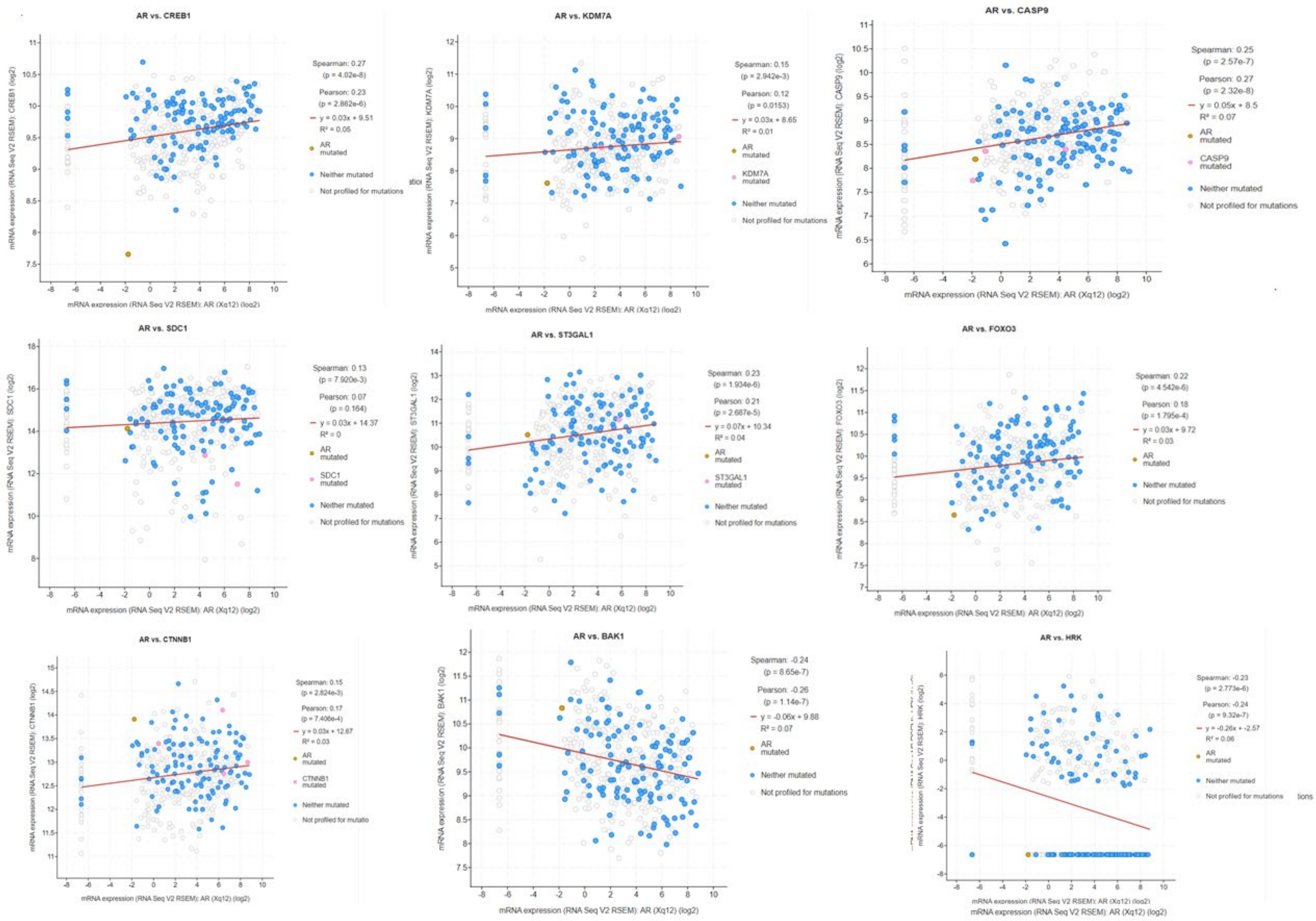

Supplementary Figure 5. Kaplan-Meier curves of disease free and overall survival of various AR-regulated transcripts were obtained from GEPIA2.

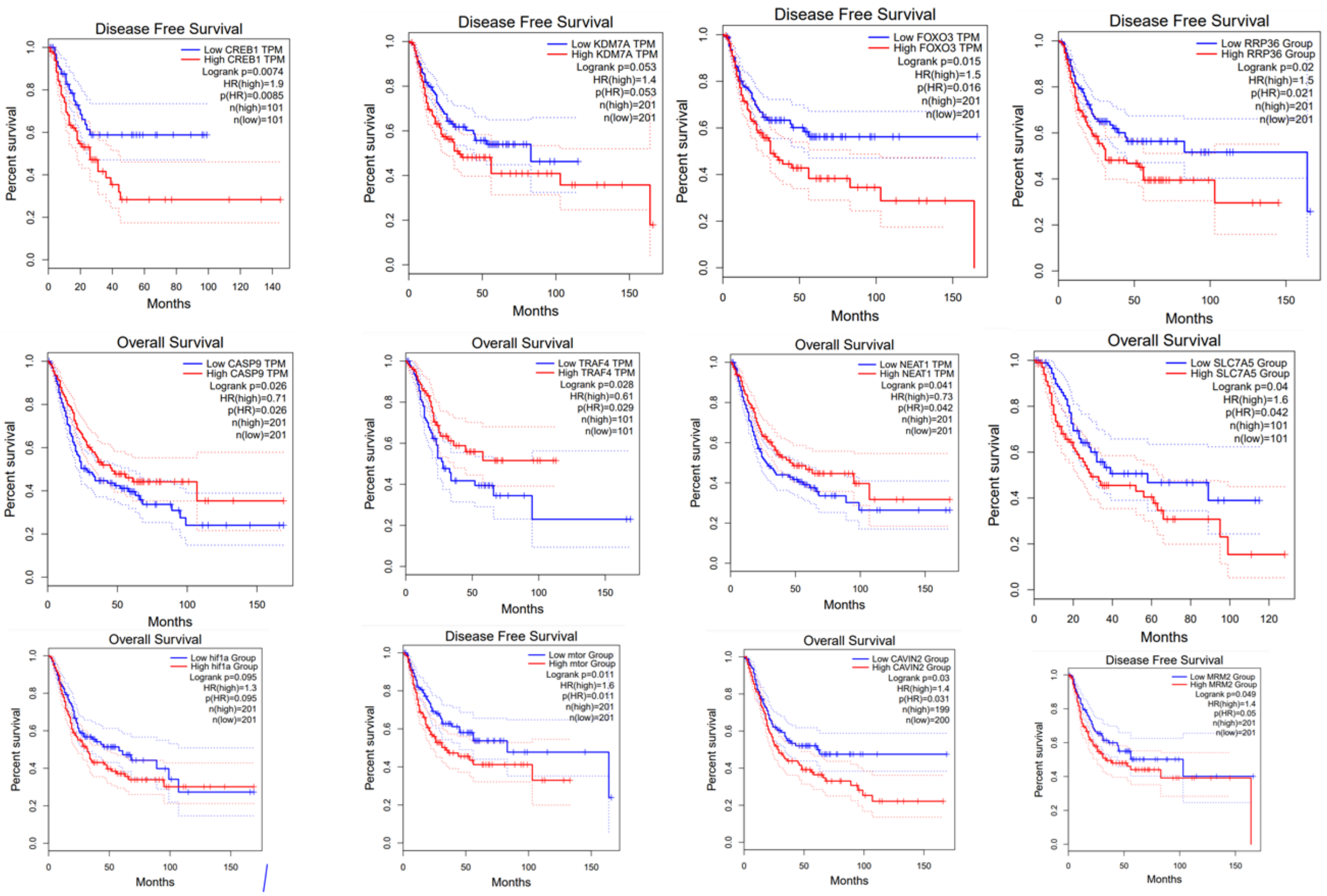

Supplementary Figure 6. Sottnik et al. identified 96 genes with identified AR-binding sites (column 1) that were AR-responsive (column 2). Comparative analysis of our data with the 96 gene set identified genes that were regulated by AR-v19 (column 3), T-AR (column 4), FL-AR (column 5) or AR-v19 overexpression (O-E AR-v19; column 6). Genes that are inversely regulated by R1881 and AR depletion are highlighted in yellow. Transcripts that are identically regulated by R1881 and AR-v19 overexpression are highlighted in green.

Chip-seq/ AR responsive	Expression change post-1881 stimulation	AR-v19 k/d	T-AR k/d	FL-AR k/d	O-E AR-v19
ADM	Up	up			up
AFAP1L1	Up	down			
ANGPTL4	Up	up			
ANK2	Up	down			
ARHGEF2	Up	down			
ARNTL2	Down				
ASAP2	Up				
ATP1A1	Up				
B3GNT5	Up	up			
BACH1	Up	up			
BAG3	Up	up			up
C10orf90	Up	up			up
CA12	Up	up			down
CAV2	Up	up	up		
CCL5	Up	up		down	
CD109	Up	up			
CD44	Down	down			up
CDC42EP3	Up	up			
CDKN1A	Up				down
COL13A1	Down				
COPS8	Up				
CRISPLD2	Up	up			up
DHCR24	Up				
DKK3	Up				
DLG1	Up				
DNER	Up	down		down	up
DUSP1	Up	up			
EPAS1	Up	up			
ERRFI1	Up	up			down
ETS1	Up				
FADS1	Up	down			
FAT4	Up	up		up	
FER	Up	up			
FHL1	Up				down
FKBP5	Up	down			
FNDC3B	Up	up			
FTH1	Up	up			
GADD45A	Up	up			
GLIS3	Up	up			up
IGF2BP3	Down				
IL1R1	Up				

KANK1	Up				
KCNMA1	Up	up			down
KLF7	Up	up	up		down
METTL7A	Up				
MICAL2	Up				
MXI1	Up	up			down
NDRG1	Up	up	up	up	down
NEDD4	Up	up			
NNMT	Up				
NR1D2	Down				
NR5A2	Up				
NRG1	Down	down			
NSG1	Up				
P4HA2	Up	up			
P4HA3	Up				
PAG1	Up				
PALLD	Down				
PHACTR2	Up	up	up		
PLIN2	Up				
PLOD2	Up	up		down	
PPP1R3C	Up	up			down
PPP2CB	Up				
PRKCH	Up				
PXYLP1	Up	up			
RERE	Up	up			down
RTN4RL1	Up				
SEC14L2	Up	up			
SH3BP4	Up	up			
SH3KBP1	Down	down			down
SH3PXD2A	Up				
SH3RF3	Up	up			
SLC26A2	Up				
SLC39A14	Up	down			down
SMOC1	Up				
SOCS2	Up				
SOD2	Up				
SQRDL	Up				
SRGN	Up		down	down	up
STEAP3	Up	up			down
STXBP6	Up				
SYNJ2	Down	up			
TBC1D8	Up				
TM9SF1	Up				
TMEM189	Up	up			
TMEM189-UBE2V1	Up	up			
TMEM44	Up	up			
TNC	Down	down			
TNFAIP3	Up				

TRIM16	Up	up	down
TRIM16L	Up	up	
TRIM9	Up	up	down
TSC22D3	Up	down	
USP43	Up	up	up
XRCC4	Down		
ZCCHC7	Down	up	
ZDHHC8	Up		

Supplementary Figure 7. Uncropped images from Figures 2,3 4,5, 6, 7. Top row, from let to right: Fig. 2A, Fig. 3A, Fig 4A., second row, Fig. 4F.

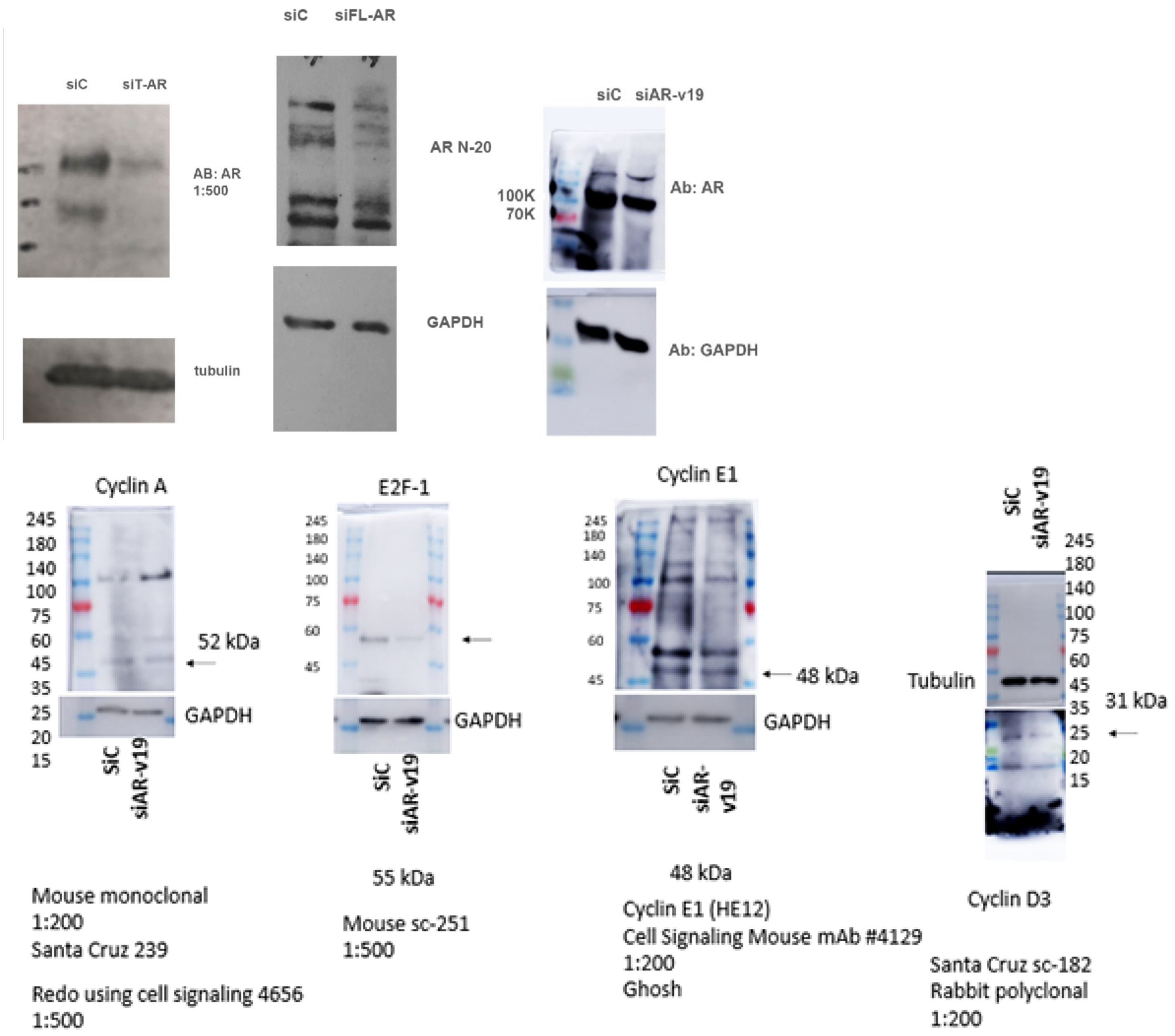

Supplementary Figure 7 con't. Top row from left to right Fig 5A, Fig. 6;, bottom row Fig 7G.

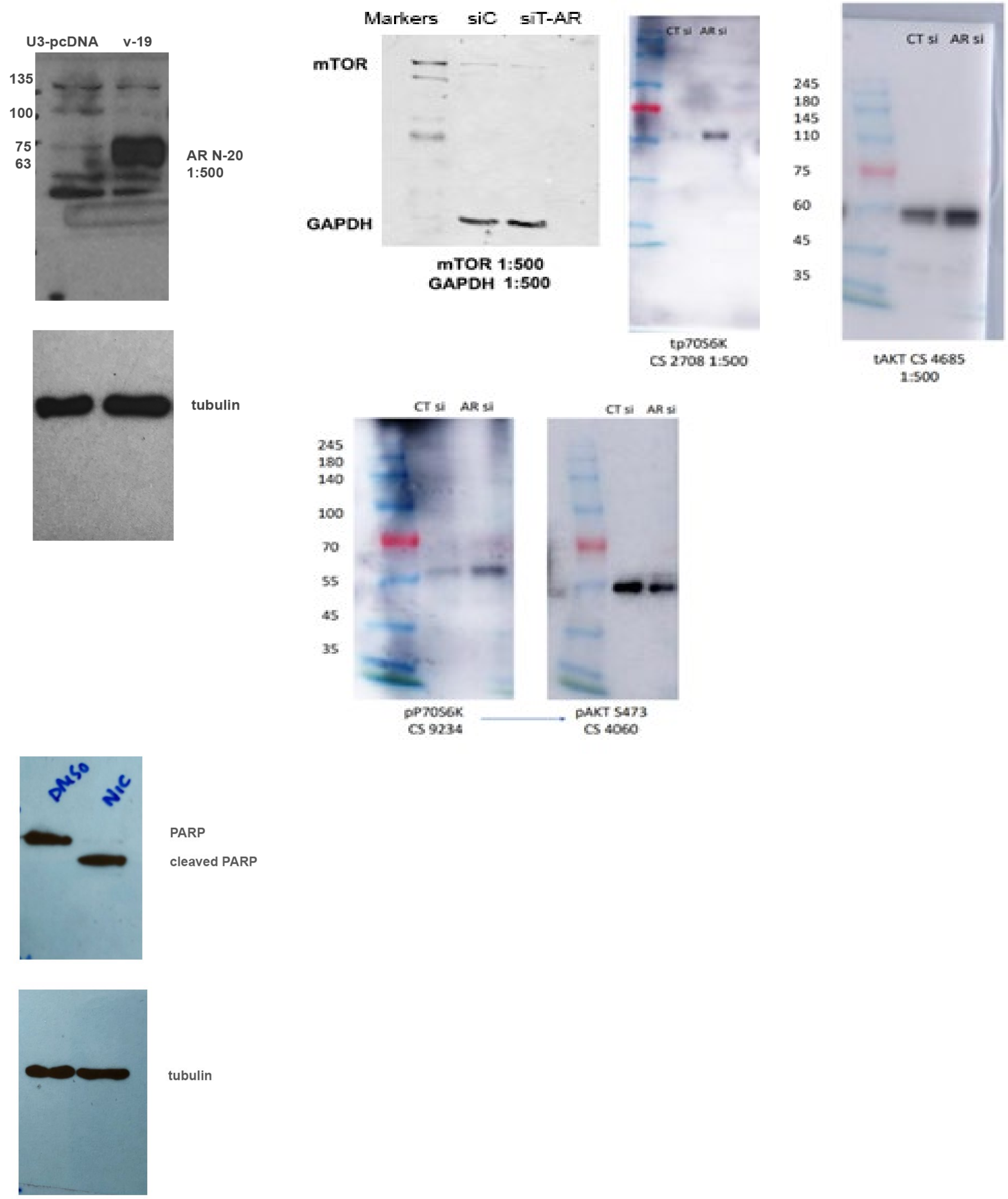

Supplementary Table.

Contents

total AR transcriptome
uique to total AR k/d
LBD transcriptome
AR-v19 transcriptome
AR-V19 top 5 pathwyas
AR-V19 top 250
altered on AR-v19 overexpression
inv. Regulated on V19 overexpression vs V19 k/d

lncRNA total AR k/d
singularly listed totla AF k/d
targets of total AR k/d
lncRNA LBD k/d
targets of LBD k/d
lncRNA V19 k/d
singularly listed V19 k/d
targets of V19 k/d
lncRNA V19 overexpressed
singularly listed v19 overexpressed
targets of V19 overexpressed
inversly reg v19 overexpressed vs V19 k/d
